# Supplementary material for: Response Type and Host Species may be Sufficient to Predict Dose-Response Curve Shape for Adenoviral Vector Vaccines
Source: Vaccines (Basel). 2020 Mar 30;8(2):155. doi: 10.3390/vaccines8020155 (PMC7349762; doi:10.3390/vaccines8020155)
Supplement: Supplementary file 1 [file vaccines-08-00155-s001.pdf]

Supplementary Figures S1

Figures are arranged hierarchically by response type, vector species, host species and route of administration, and paper number, in that order. For each dataset the time since inoculation is given, along with the vector if there were more than one used in that paper. For each dataset two plots are shown, with the left plot including the calibrated saturating curve and the right plot the calibrated peaking curve. The AIC is included for each. Blue dots represent the mean response for each dosing group. Black dots represent individual responses or upper and lower confidence intervals, depending on the data availability for that dataset. The x axis for each plot is the log10 of the given dose, and the y axis is this recorded response for that dataset, as given in the paper that the dataset was taken from.

Response Type: Antibody

Vector Species: B

Host Species: Mouse

Route of Administration: IM

Paper 578: Ad35 and ad26 vaccine vectors induce potent and cross-reactive antibody and T-cell responses to multiple filovirus species

Day 28 - Ad35 Zaire

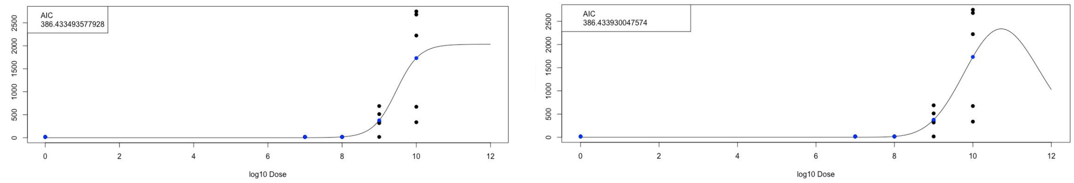

Day 28 - Ad35 Angola

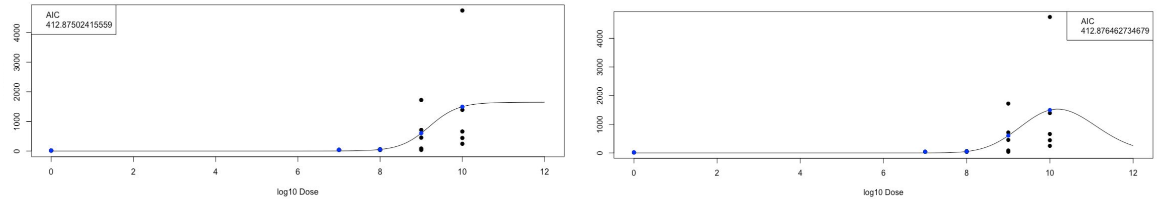

Day 28 - Ad35 S/G

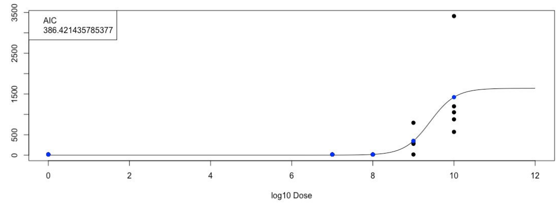

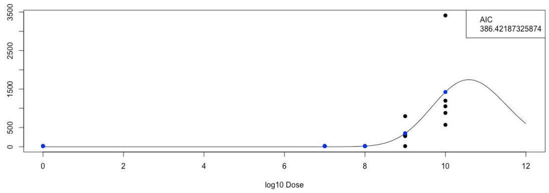

Day 28 - Ad35 Ravn

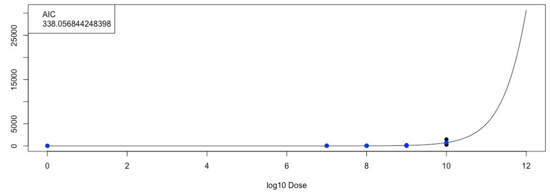

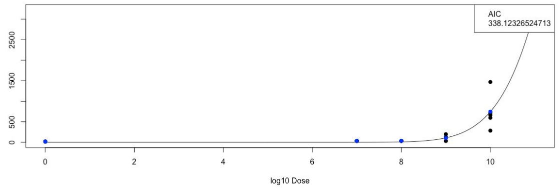

Day 28 - Ad35 I.C

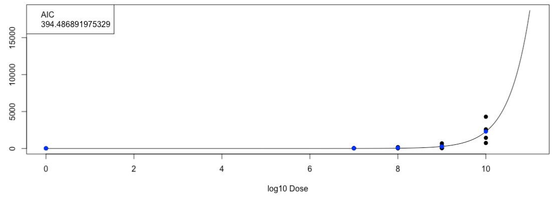

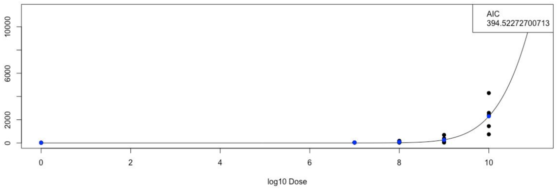

Paper 1269: Increased immunogenicity of recombinant Ad35-based malaria vaccine through formulation with aluminium phosphate adjuvant

Day 56

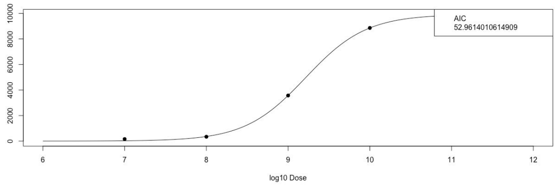

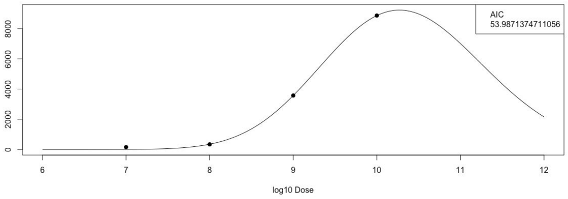

Paper 1492: Immunogenicity and Protection of a Recombinant Human Adenovirus Serotype 35-Based Malaria Vaccine against Plasmodium yoelii in Mice

Day 14

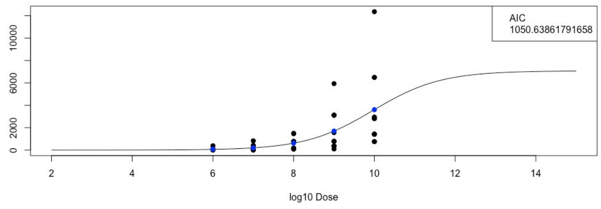

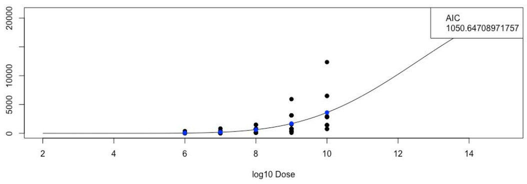

Host Species: Human

Route of Administration: IM

Paper 441: A phase 1b randomized, controlled, double-blinded dosage-escalation trial to evaluate the safety, reactogenicity and immunogenicity of an adenovirus type 35 based circumsporozoite malaria vaccine in Burkinabe healthy adults 18 to 45 years of age

Day 28

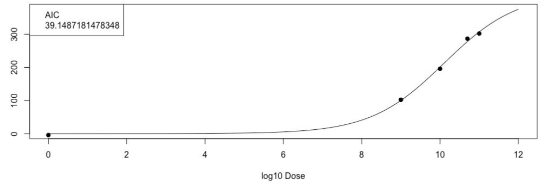

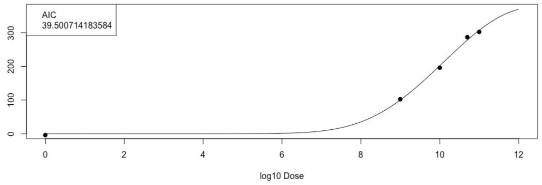

Paper 467: Randomized, placebo-controlled trial to assess the safety and immunogenicity of an adenovirus type 35-based circumsporozoite malaria vaccine in healthy adults

Day 60

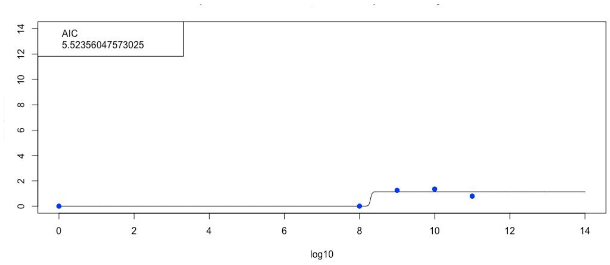

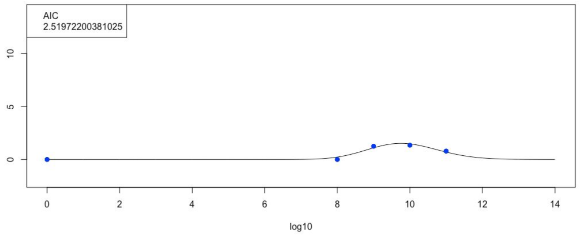

Paper 633: A phase I double blind, placebo-controlled, randomized study of a multigenic HIV-1 adenovirus subtype 35 vector vaccine in healthy uninfected adults

Day 28

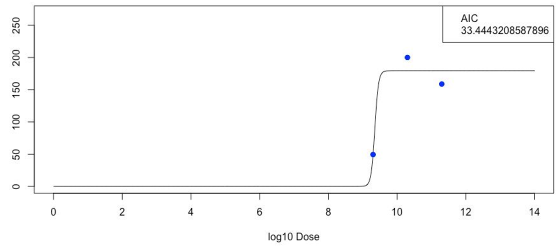

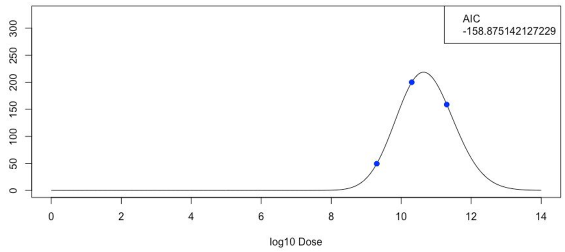

Vector Species: C

Host Species: Rabbit

Route of Administration: IM

Paper 744: A novel alphavirus replicon-vectored vaccine delivered by adenovirus induces sterile immunity against classical swine fever

Day 28

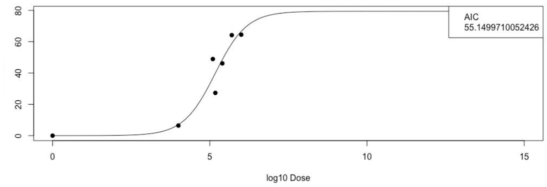

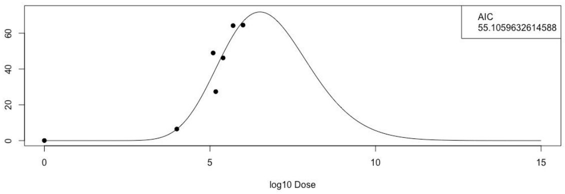

Host Species: Mouse

Route of Administration: IM

Paper 461: Beta-defensin 2 enhances immunogenicity and protection of an adenovirus-based H5N1 influenza vaccine at an early time

Day 7

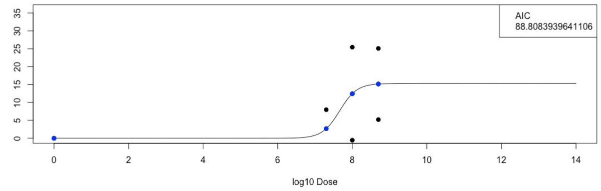

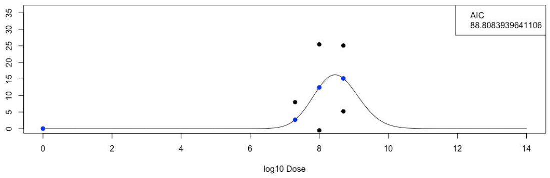

Day 14

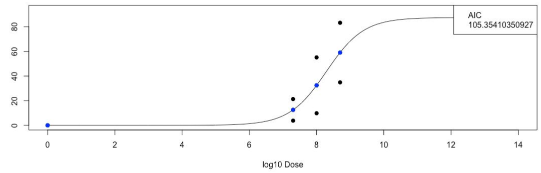

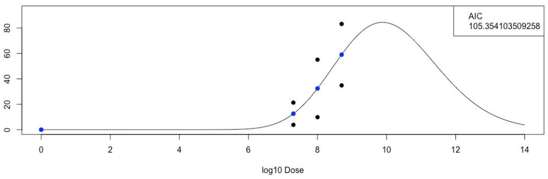

Paper 574: Recombinant adenovirus expressing type Asia1 foot-and-mouth disease virus capsid proteins induces protective immunity against homologous virus challenge in mice

Day 7

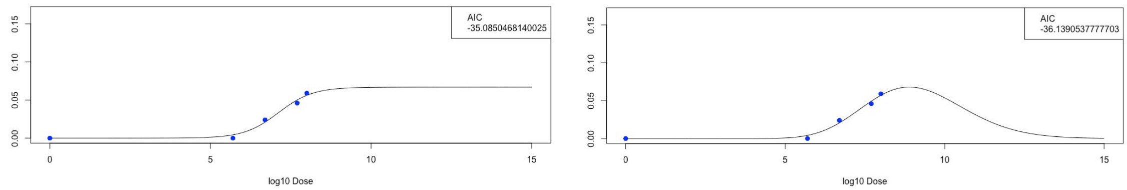

Day 21

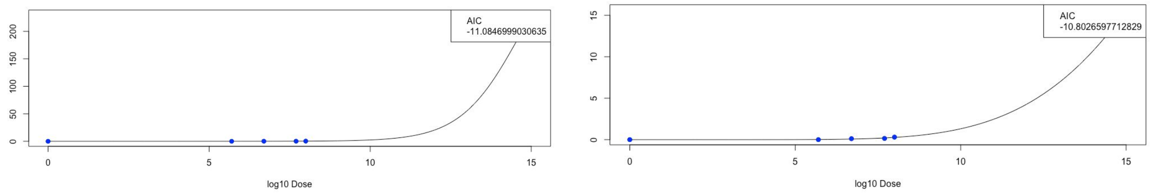

Day 35

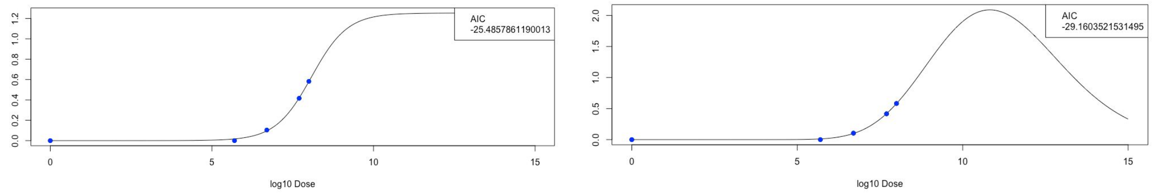

Day 49

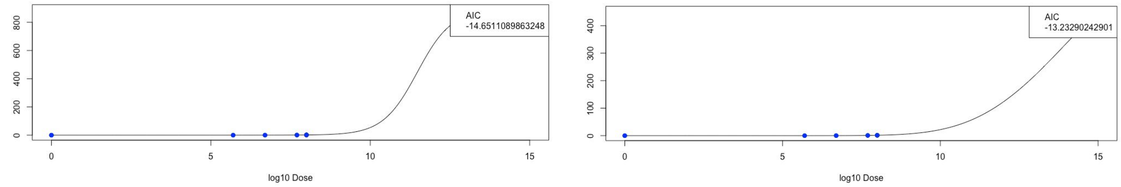

Day 63

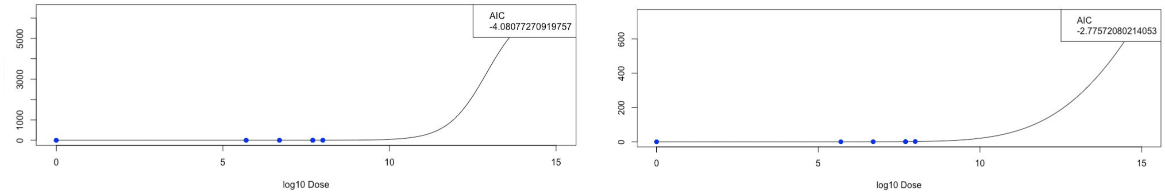

Day 77

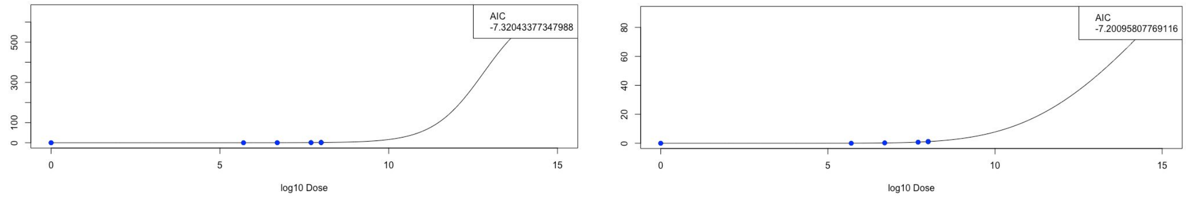

Day 91

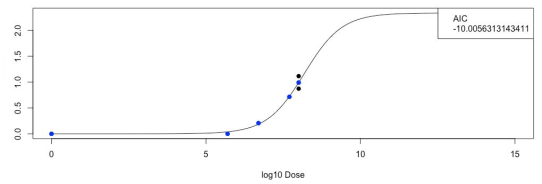

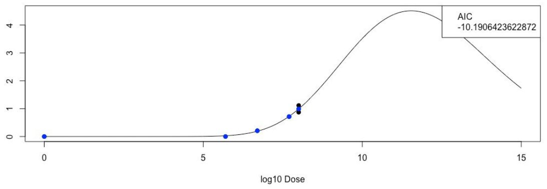

Day 119

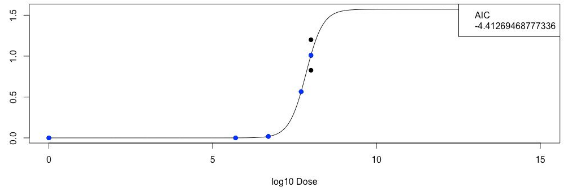

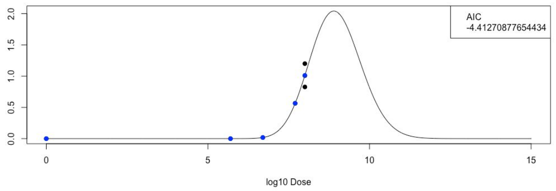

Day 147

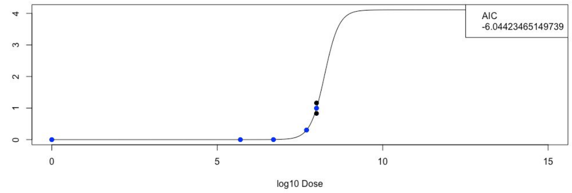

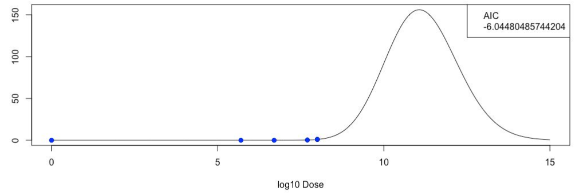

Day 161

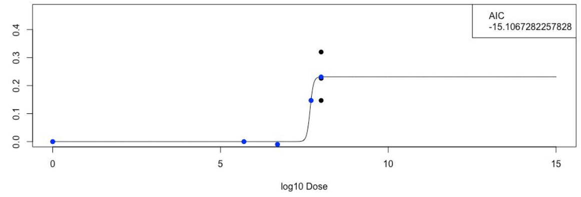

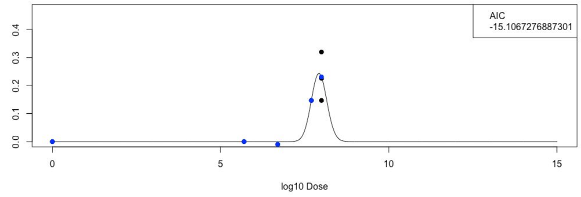

Paper 1492: Immunogenicity and Protection of a Recombinant Human Adenovirus Serotype 35-Based Malaria Vaccine against Plasmodium yoelii in Mice

Day 14

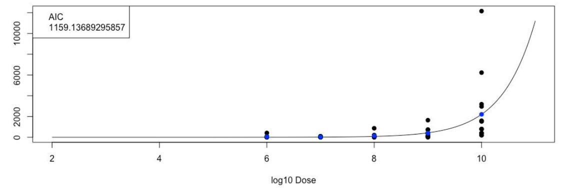

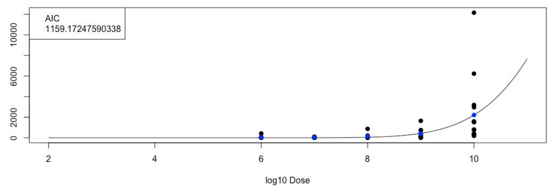

Paper 2531: Isogenic adenoviruses type 5 expressing or not expressing the E1A gene: efficiency as virus vectors in the vaccination of permissive and non-permissive species

Day 10 Ad-gD

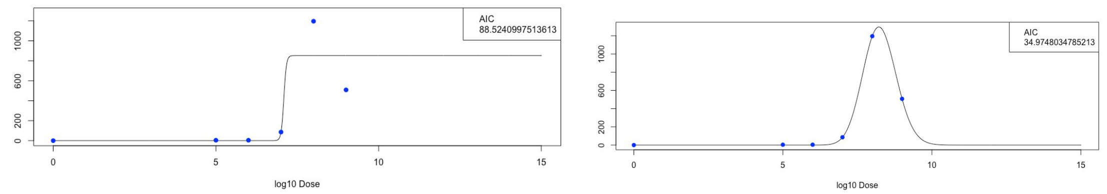

Day 10 Ad-gD-E1A

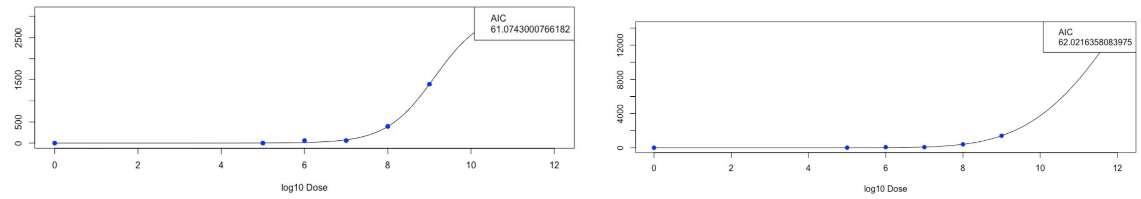

Route of Administration: SQ

Paper 936: A Candidate H1N1 Pandemic Influenza Vaccine Elicits Protective Immunity in Mice

Day 35 - Wildtype

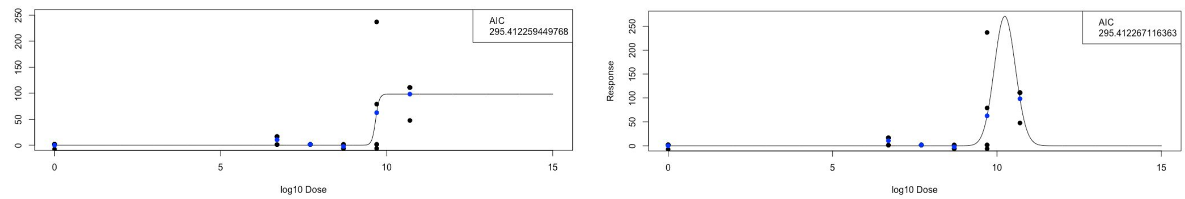

Day 35 - Codon Optimized

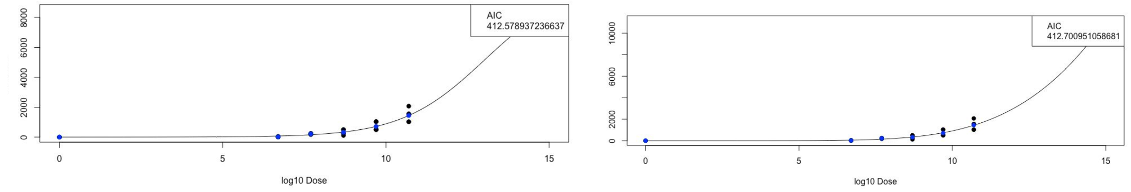

Host Species: Human

Route of Administration: IM

Paper 140: Use of ChAd3-EBO-Z Ebola virus vaccine in Malian and US adults, and boosting of Malian adults with MVA-BN-Filo: a phase 1, single-blind, randomised trial, a phase 1b, open-label and double-blind, dose-escalation trial, and a nested, randomised, double-blind, placebo-controlled trial

Day 28

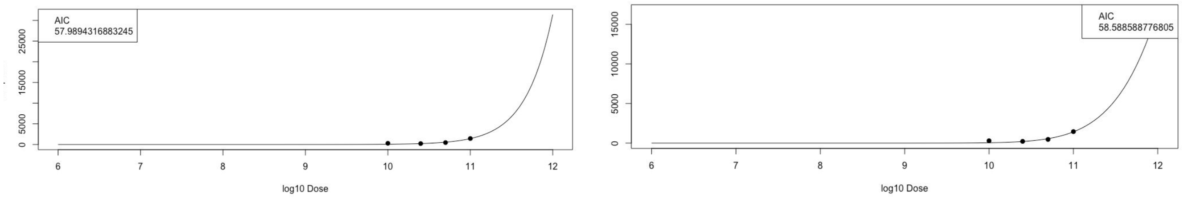

Paper 249: A Monovalent Chimpanzee Adenovirus Ebola Vaccine Boosted with MVA

Day 14

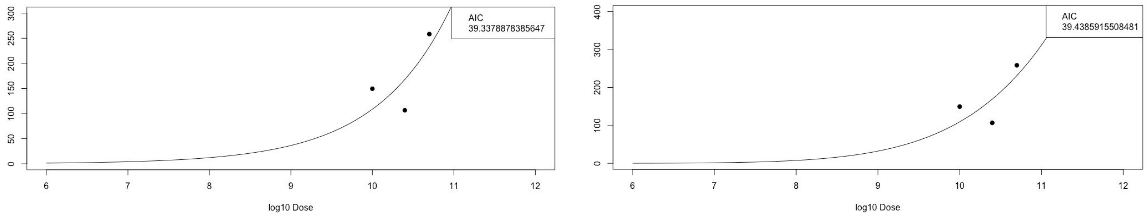

Day 28

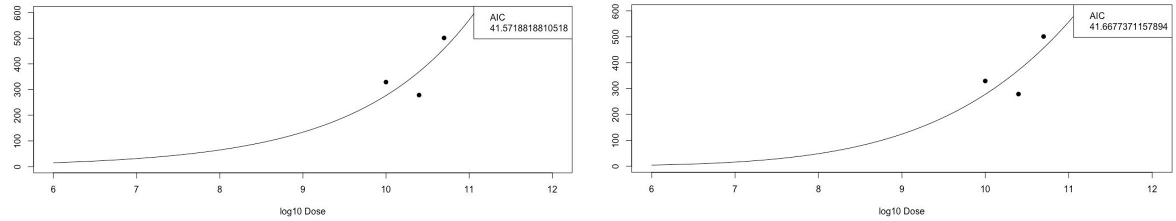

Day 180

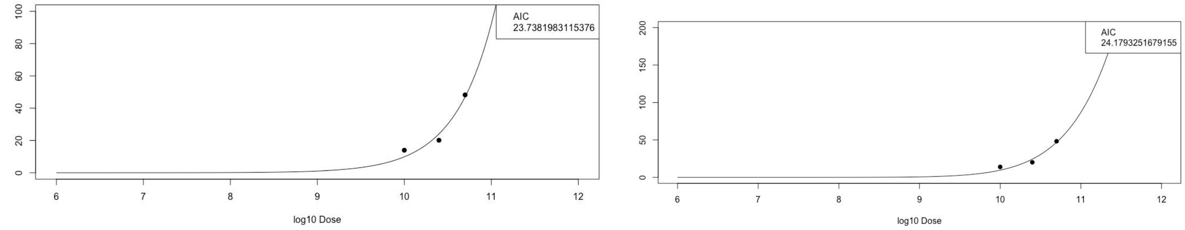

Host Species: Monkey

Route of Administration: IM

Paper 1877:Comparative immunogenicity in rhesus monkeys of DNA plasmid, recombinant vaccinia virus, and replication-defective adenovirus vectors expressing a human immunodeficiency virus type 1 gag gene

Day 28

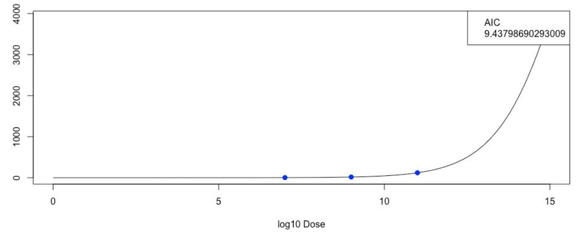

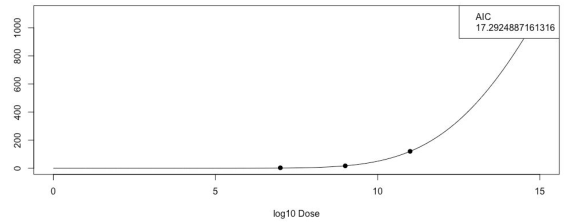

Day 56

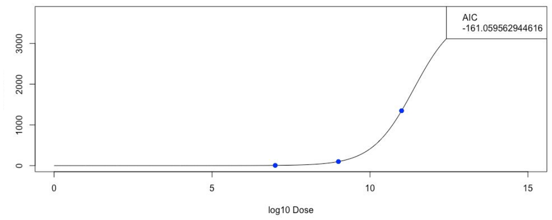

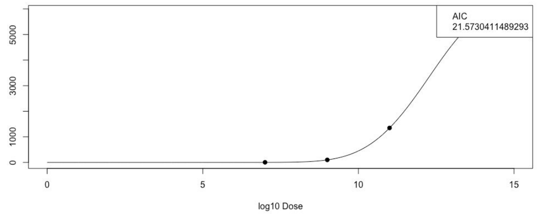

Day 84

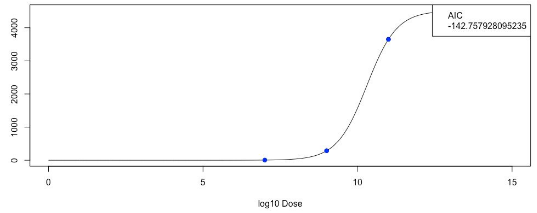

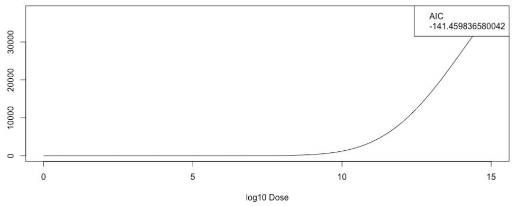

Day 112

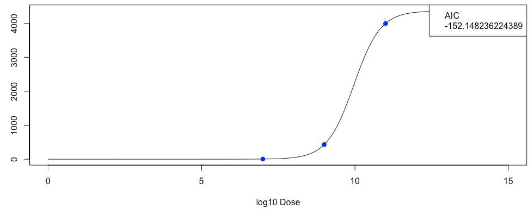

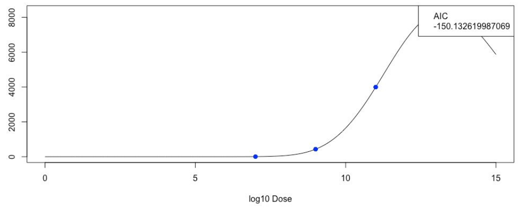

Day 140

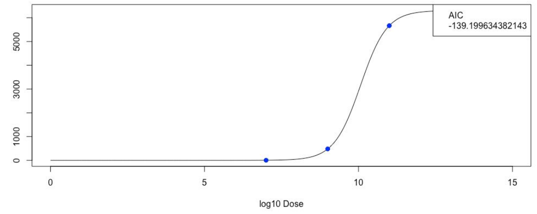

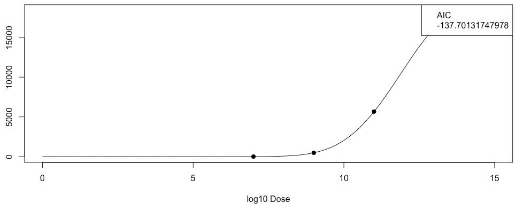

Host Species: Rat

Route of Administration: IM

Paper 2531: Isogenic adenoviruses type 5 expressing or not expressing the E1A gene: efficiency as virus vectors in the vaccination of permissive and non-permissive species

Day 10 Ad-gD

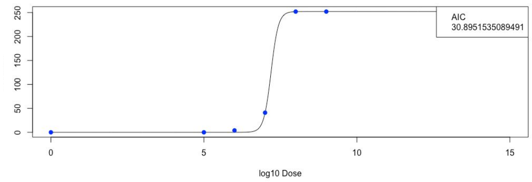

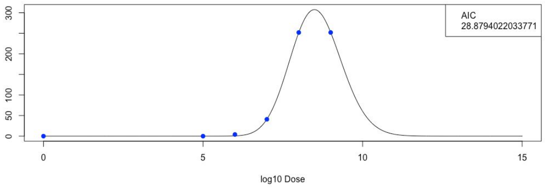

Day 10 Ad-gD-E1A

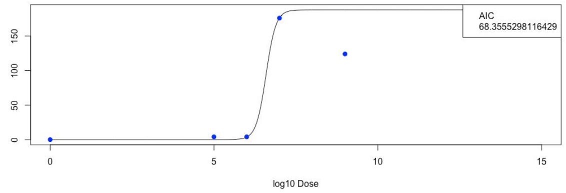

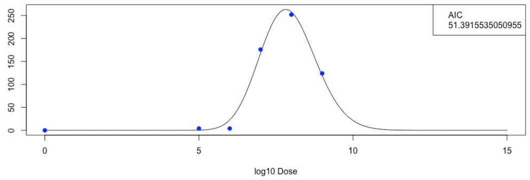

Vector Species: D

Host Species: Mouse

Route of Administration: IM

Paper 578: Ad35 and ad26 vaccine vectors induce potent and cross-reactive antibody and T-cell responses to multiple filovirus species

Day 28 - Ad26 Zaire

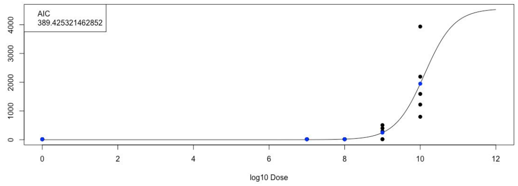

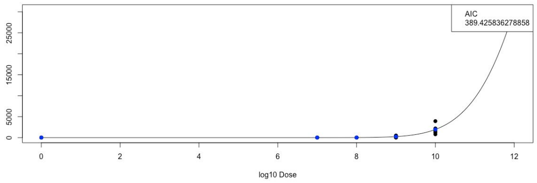

Day 28 - Ad26 Angola

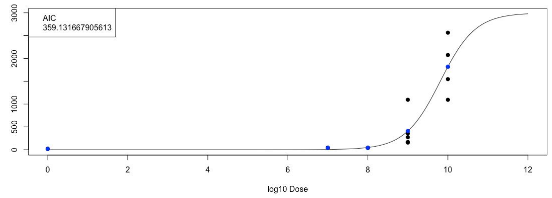

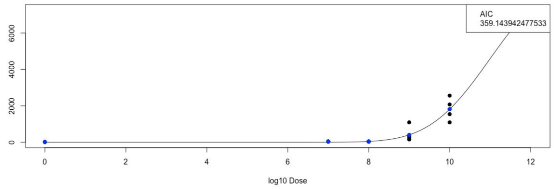

Day 28 - Ad26 S/G

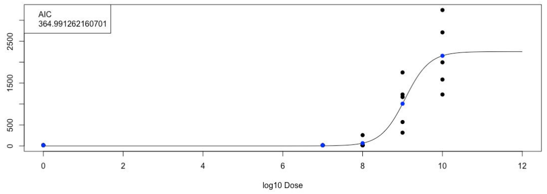

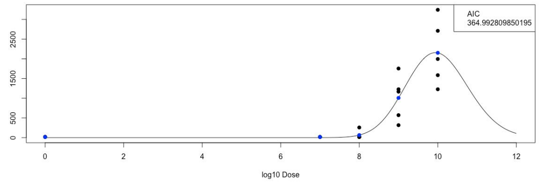

Day 28 - Ad26 Ravn

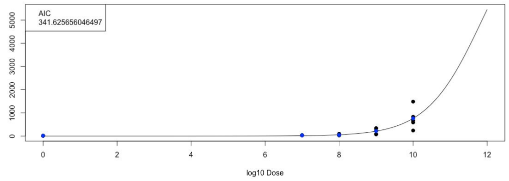

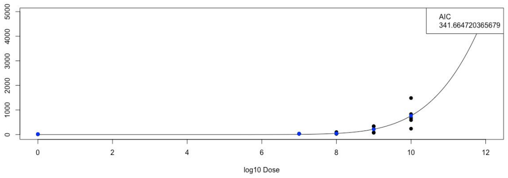

Day 28 - Ad26 I.C

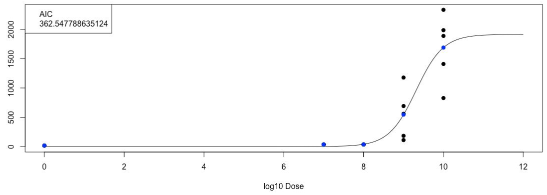

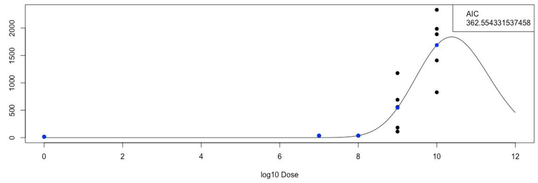

Host Species: Human

Route of Administration: IM

Paper 594: First-in-human evaluation of the safety and immunogenicity of a recombinant adenovirus serotype 26 HIV-1 Env vaccine (IPCAVD 001)

Day 14

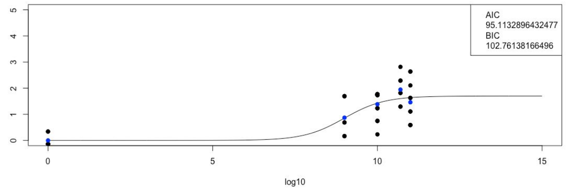

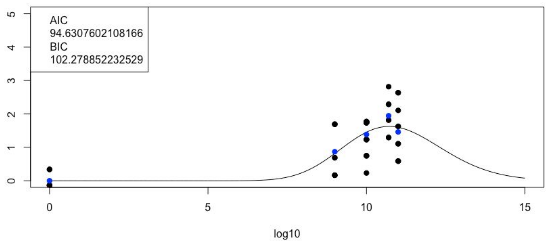

Vector Species: E

Host Species: Mouse

Route of Administration: IM

Paper 1539: Induction of Protective Immunity to Anthrax Lethal Toxin with a Nonhuman Primate Adenovirus-Based Vaccine in the Presence of Preexisting Anti-Human Adenovirus Immunity

Day 14 - Anti PA

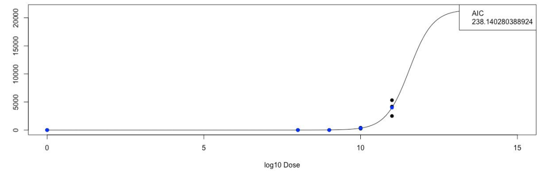

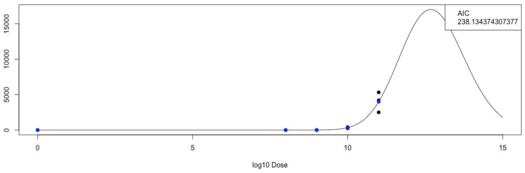

Day 28 - Anti PA

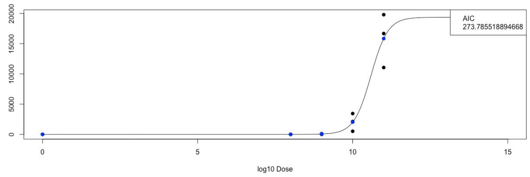

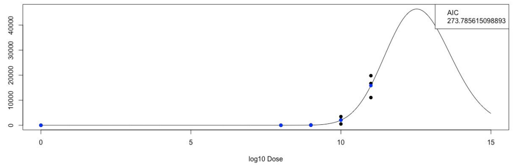

Day 42 - Anti PA

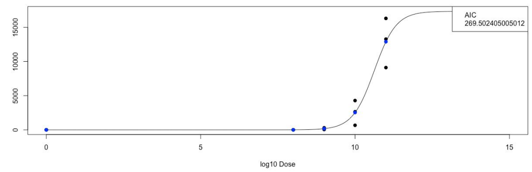

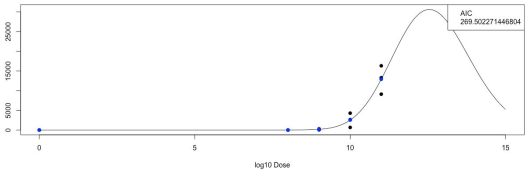

Day 14 - Anti LT

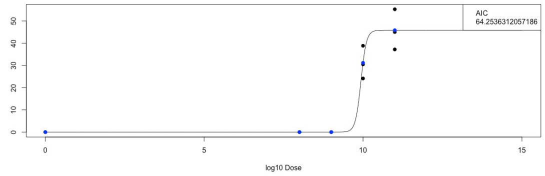

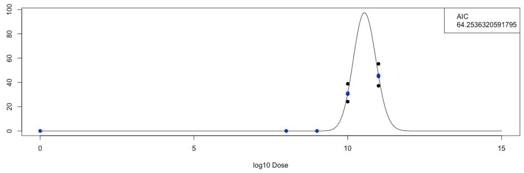

Day 28 - Anti LT

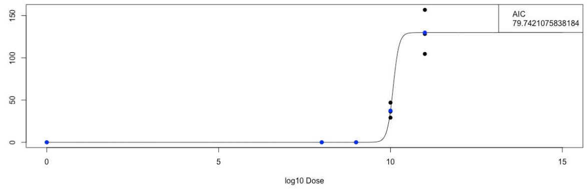

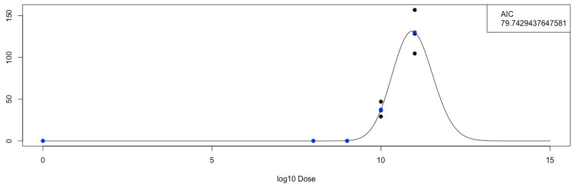

Day 42 - Anti LT

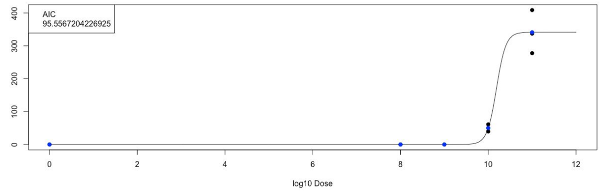

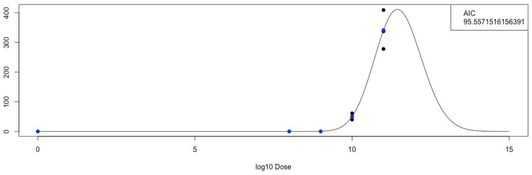

Paper 2919: A prime-boost immunization regimen based on a Simian Adenovirus 36 vectored multi-stage malaria vaccine induces protective immunity in mice.

Day 20

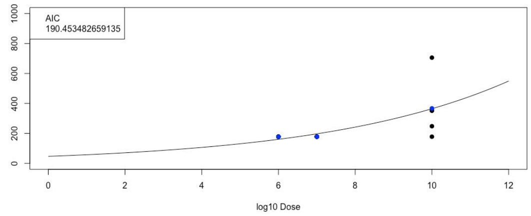

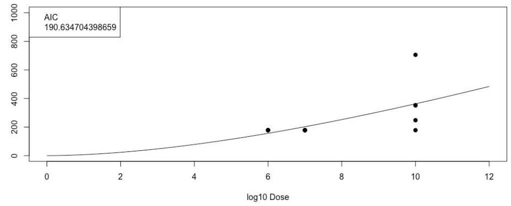

Host Species: Human

Route of Administration: IM

Paper 417: Clinical assessment of a novel recombinant simian adenovirus ChAdOx1 as a vectored vaccine expressing conserved Influenza A antigens

Day 14

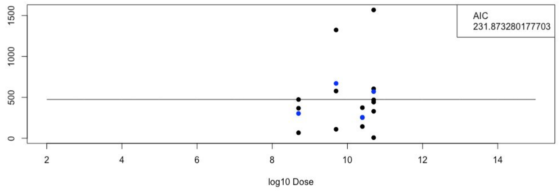

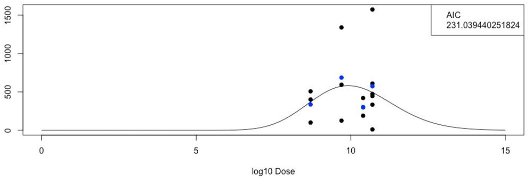

Day 21

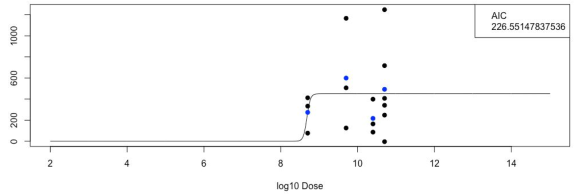

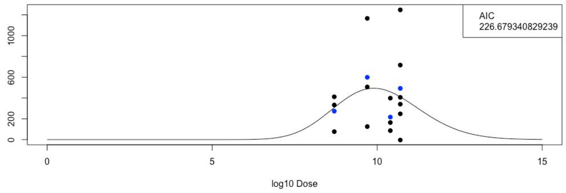

Response Type: T cell

Vector Species: B

Host Species: Mouse

Route of Administration: IM

Paper 578: Ad35 and ad26 vaccine vectors induce potent and cross-reactive antibody and T-cell responses to multiple filovirus species

Day 28 - Ad35 Zaire

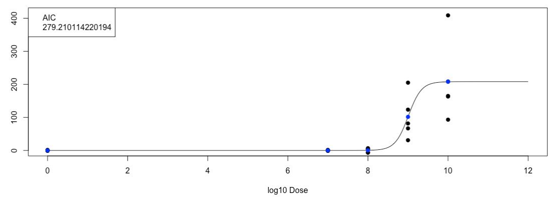

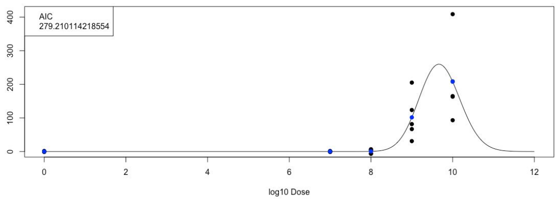

Day 28 - Ad35 Angola

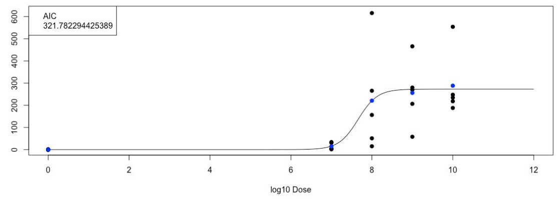

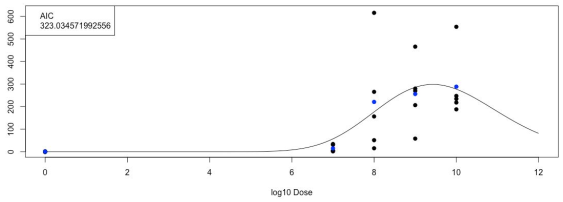

Day 28 - Ad35 S/G

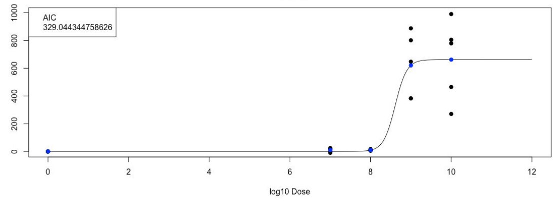

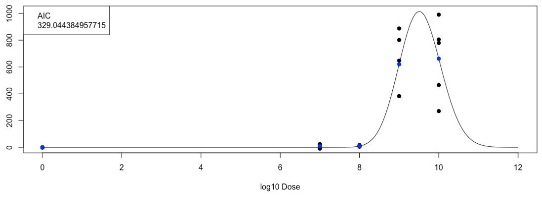

Day 28 - Ad35 Ravn

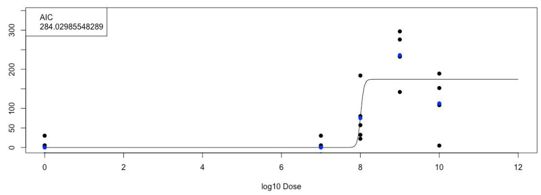

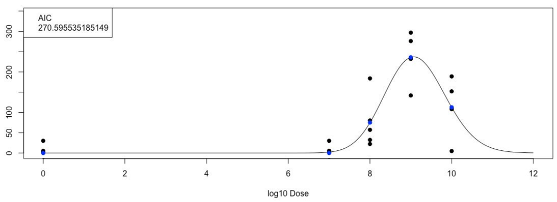

Day 28 - Ad35 I.C

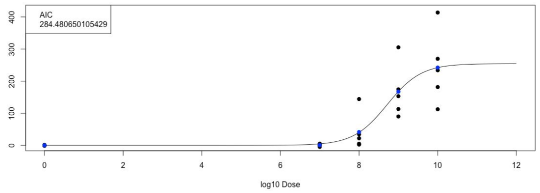

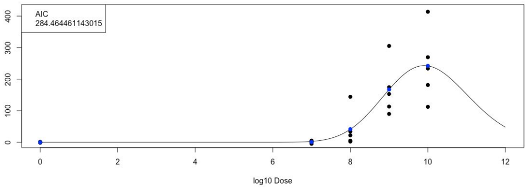

Paper 1269: Increased immunogenicity of recombinant Ad35-based malaria vaccine through formulation with aluminium phosphate adjuvant

Day 56

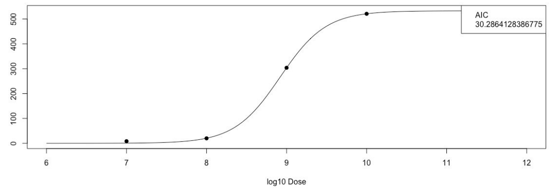

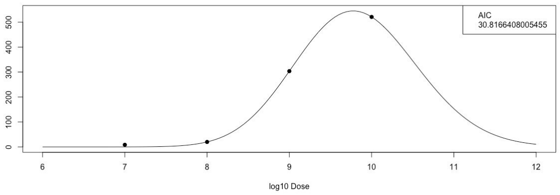

Host Species: Human

Route of Administration: IM

Paper 441: A phase 1b randomized, controlled, double-blinded dosage-escalation trial to evaluate the safety, reactogenicity and immunogenicity of an adenovirus type 35 based circumsporozoite malaria vaccine in Burkinabe healthy adults 18 to 45 years of age

Day 28

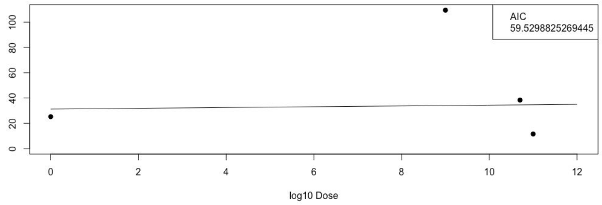

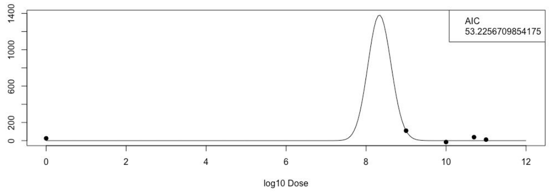

Paper 633: A phase I double blind, placebo-controlled, randomized study of a multigenic HIV-1 adenovirus subtype 35 vector vaccine in healthy uninfected adults

Day 14

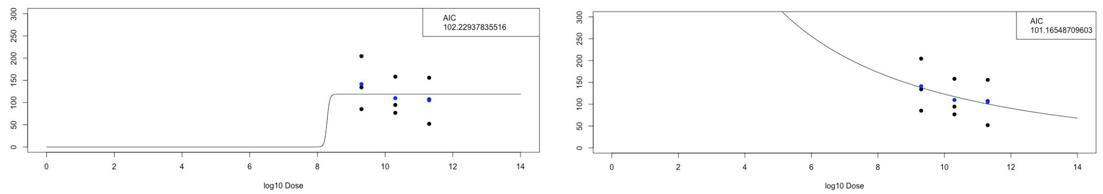

Day 28

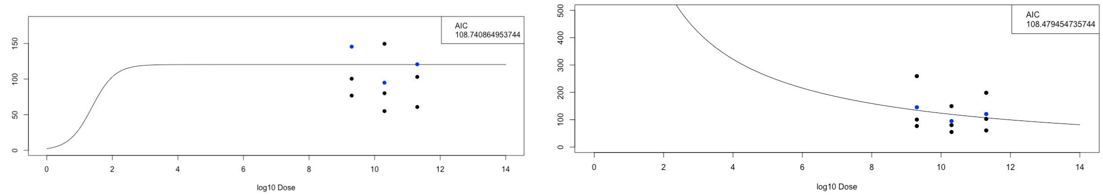

Vector Species: C

Host Species: Mouse

Route of Administration: IM

Paper 2916: Functionally inactivated dominant viral antigens of human cytomegalovirus delivered in replication incompetent adenovirus type 6 vectors as vaccine candidates.

Day 25 - Wildtype pp65

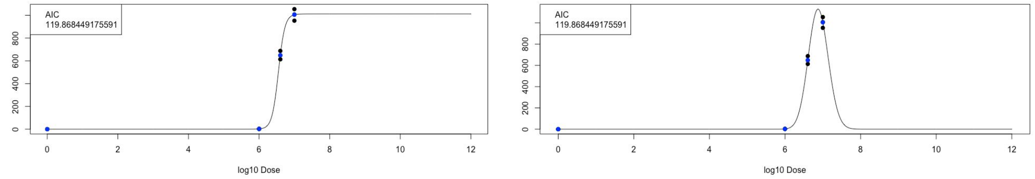

Day 25 - Modified pp65

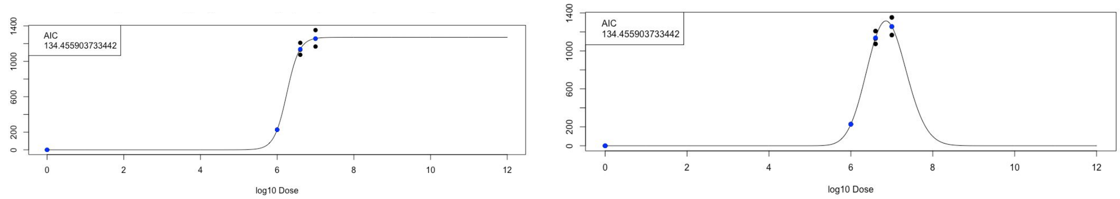

Day 25 - Wildtype Ad-IE1

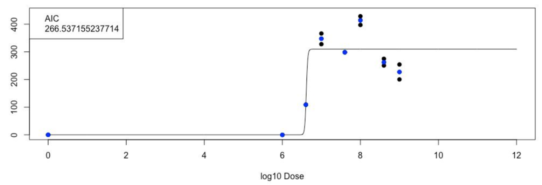

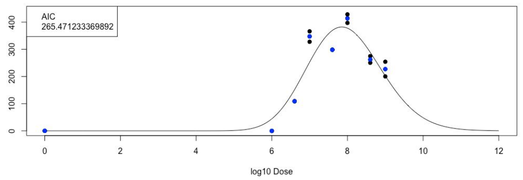

Day 25 - Modified Ad-IE1

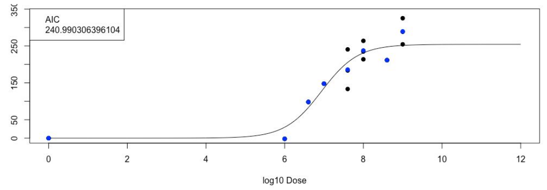

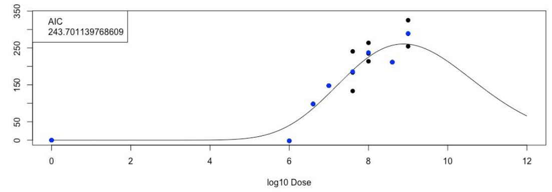

Day 25 - Wildtype Ad-IE2

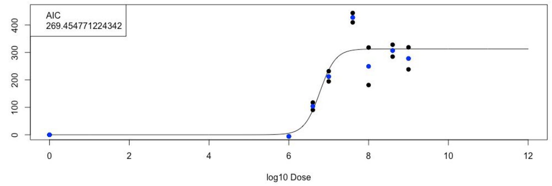

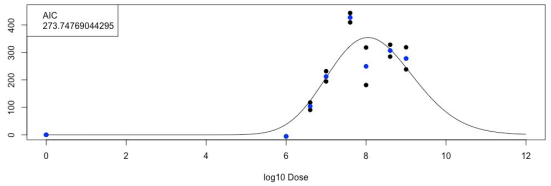

Day 25 - Modified Ad-IE1

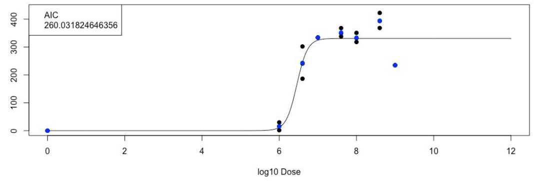

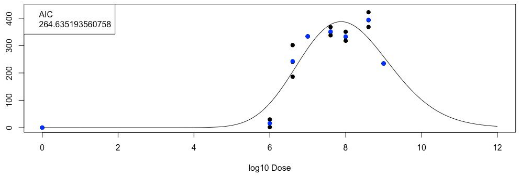

Host Species: Monkey

Route of Administration: IM

Paper 1474: A novel adenovirus type 6 (Ad6)-based hepatitis C virus vector that overcomes preexisting anti-ad5 immunity and induces potent and broad cellular immune responses in rhesus macaques

Day 8 - Ad5

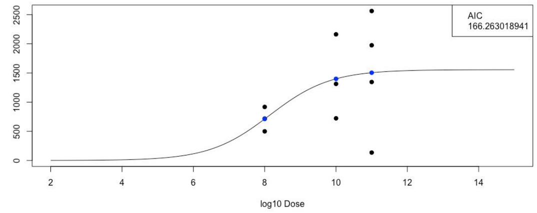

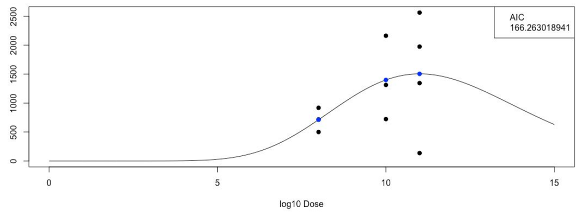

Day 24 - Ad5

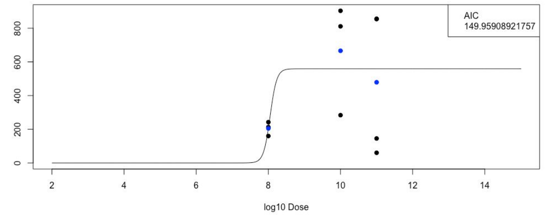

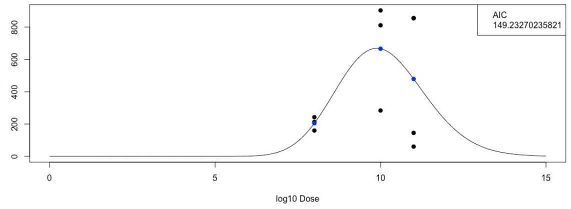

Day 8 - Ad6

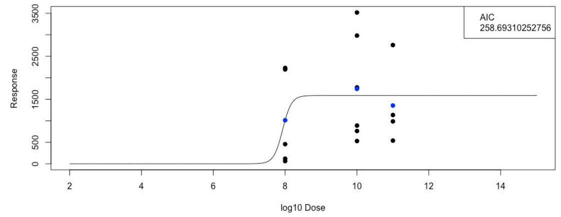

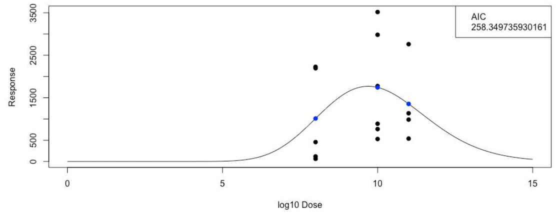

Day 24 - Ad6

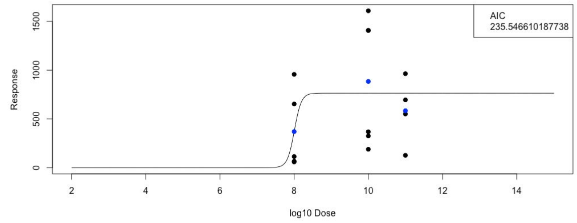

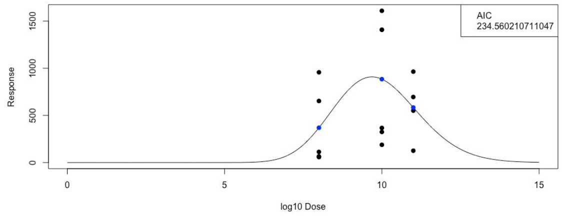

Paper 1877: Comparative immunogenicity in rhesus monkeys of DNA plasmid, recombinant vaccinia virus, and replication-defective adenovirus vectors expressing a human immunodeficiency virus type 1 gag gene

Day 28

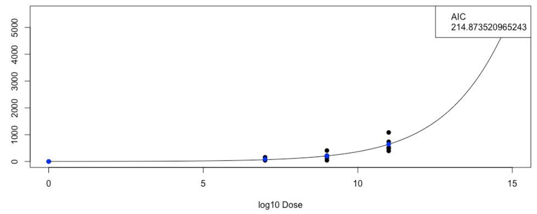

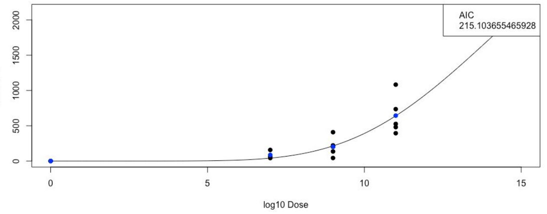

Day 168

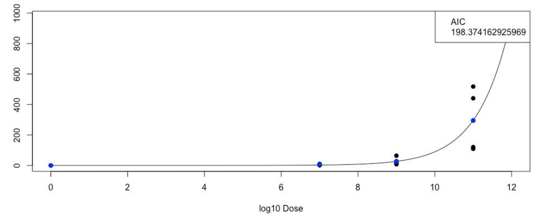

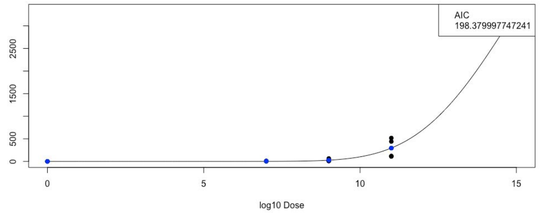

Vector Species: D

Host Species: Mouse

Route of Administration: IM

Paper 578: Ad35 and ad26 vaccine vectors induce potent and cross-reactive antibody and T-cell responses to multiple filovirus species

Day 28 - Ad26 Zaire

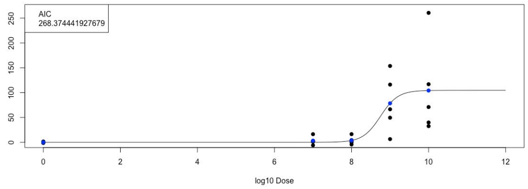

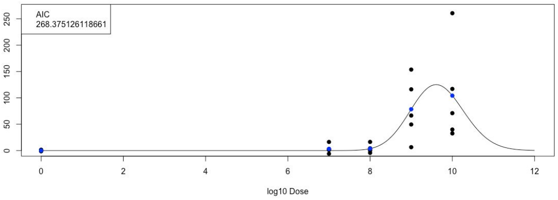

Day 28 - Ad26 Angola

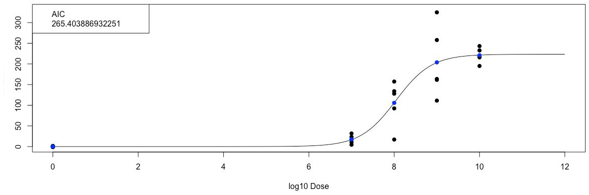

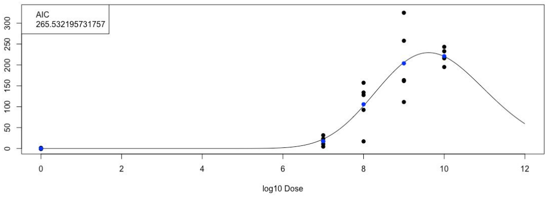

Day 28 - Ad26 S/G

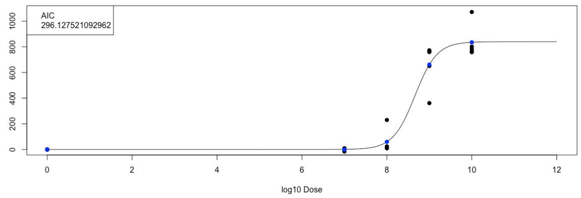

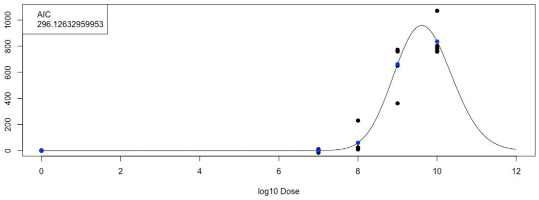

Day 28 - Ad26 Ravn

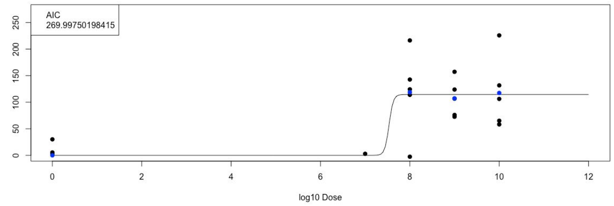

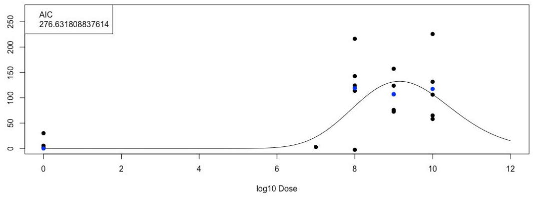

Day 28 - Ad26 I.C

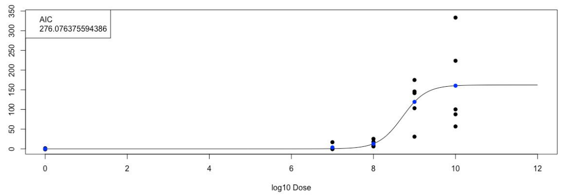

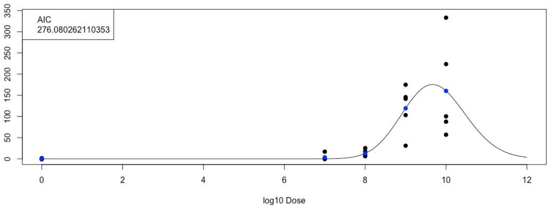

Paper 924 TLR4 Ligands Augment Antigen-Specific CD8+ T Lymphocyte Responses Elicited by a Viral Vaccine Vector

Day 14

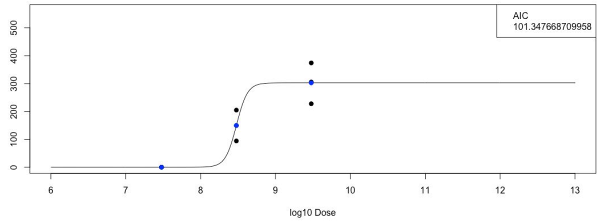

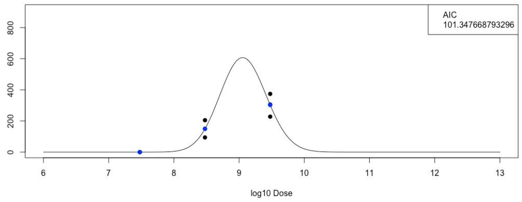

Host Species: Human

Route of Administration: IM

Paper 594: First-in-human evaluation of the safety and immunogenicity of a recombinant adenovirus serotype 26 HIV-1 Env vaccine (IPCAVD 001)

Day 14

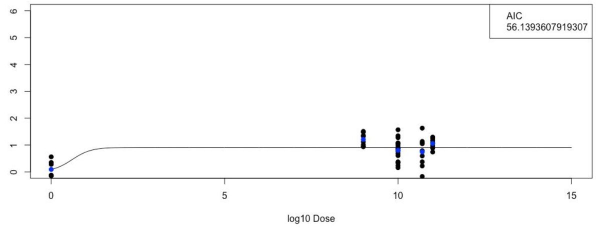

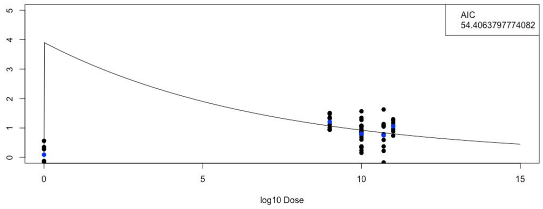

Vector Species: E

Host Species: Mouse

Route of Administration: IM

Paper 305: Characterization of T-Cell Responses to Conserved Regions of the HIV-1 Proteome in BALB/c Mice

Day 21

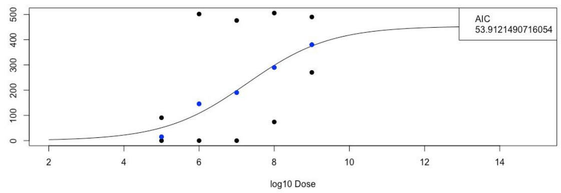

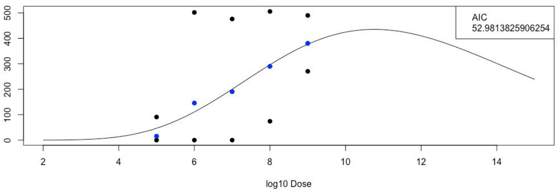

Host Species: Human

Route of Administration: IM

Paper 417: Clinical assessment of a novel recombinant simian adenovirus ChAdOx1 as a vectored vaccine expressing conserved Influenza A antigens

Day 14

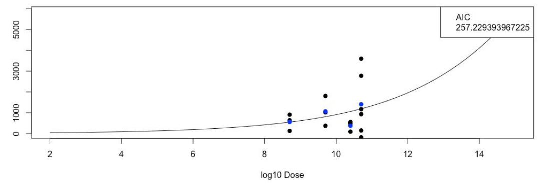

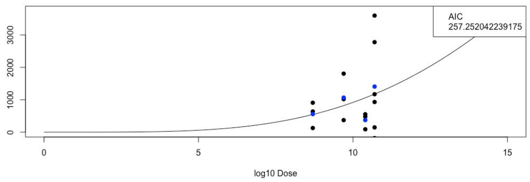

Day 21

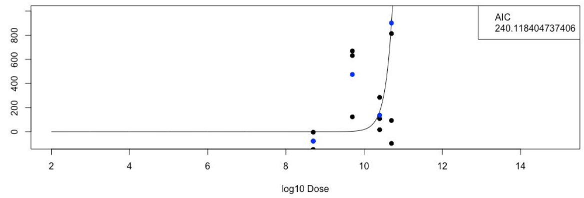

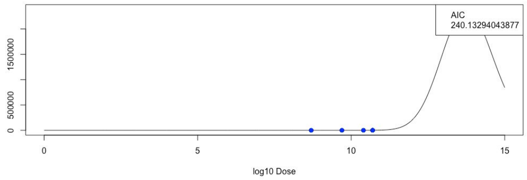

Paper 686: Clinical assessment of a recombinant simian adenovirus ChAd63: a potent new vaccine vector

Day 14

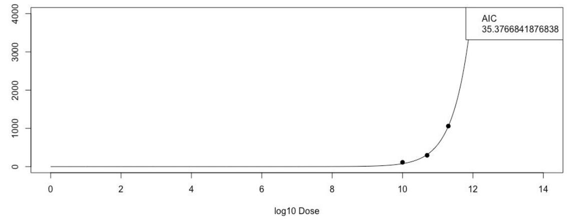

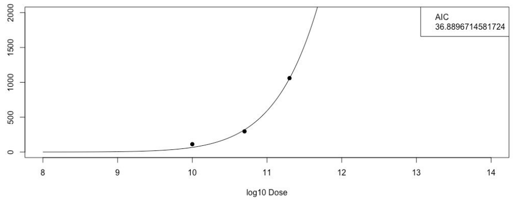

Day 21

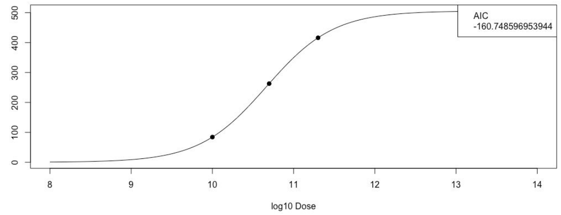

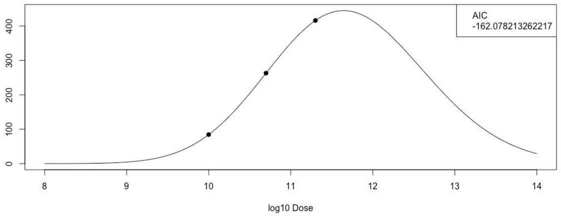

Day 90

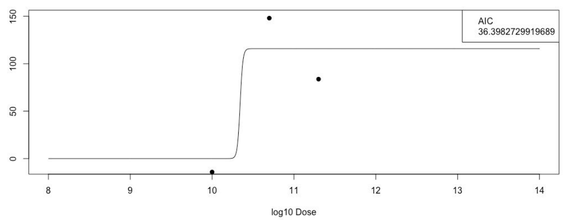

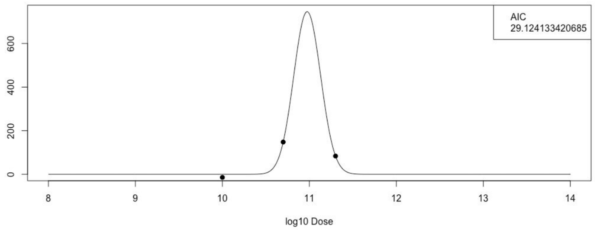

Response Type: CD4

Vector Species: B

Host Species: Mouse

Route of Administration: IM

Paper 1201: Impact of Recombinant Adenovirus Serotype 35 Priming versus Boosting of a Plasmodium falciparum Protein: Characterization of T- and B-Cell Responses to Liver-Stage Antigen 1

Day 14

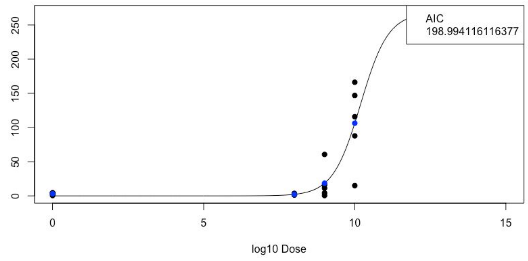

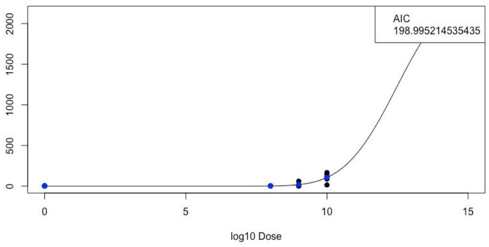

Host Species: Human

Route of Administration: IM

Paper 309: The novel tuberculosis vaccine, AERAS-402, is safe in healthy infants previously vaccinated with BCG, and induces dose-dependent CD4 and CD8T cell responses

Day 28 - Ag85A/b

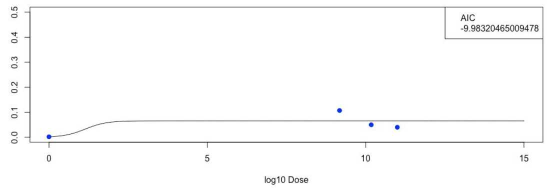

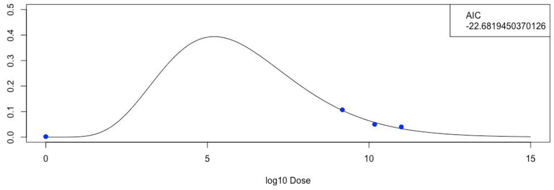

Day 28 - Tb10.4

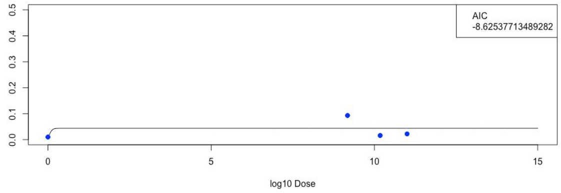

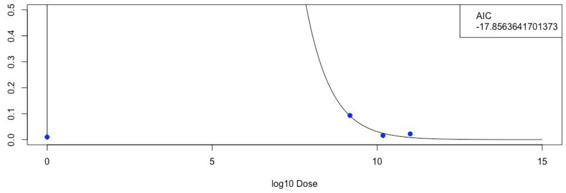

Vector Species: C

Host Species: Monkey

Route of Administration: IM

Paper 1474: A novel adenovirus type 6 (Ad6)-based hepatitis C virus vector that overcomes preexisting anti-ad5 immunity and induces potent and broad cellular immune responses in rhesus macaques

Day 8 - Ad5

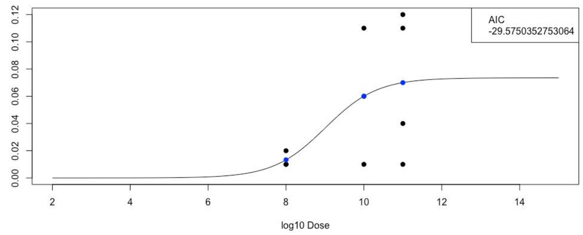

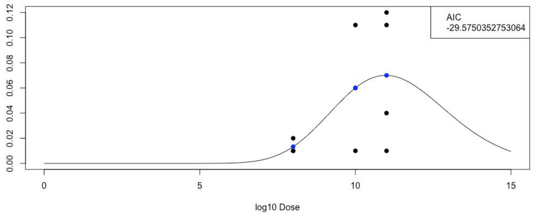

Day 8 - Ad6

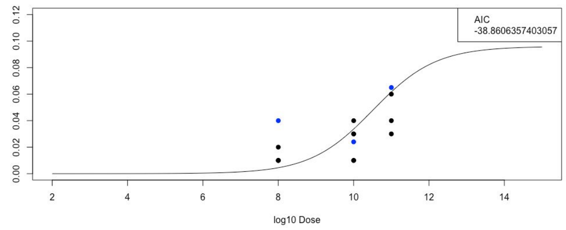

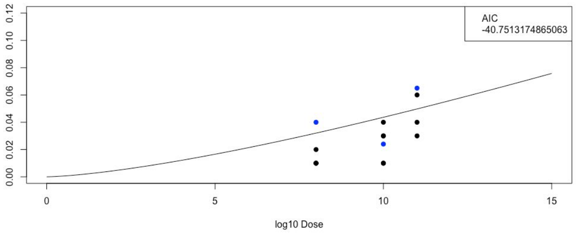

Vector Species: D

Host Species: Mouse

Route of Administration: IM

Paper 924 TLR4 Ligands Augment Antigen-Specific CD8+ T Lymphocyte Responses Elicited by a Viral Vaccine Vector

Day 14

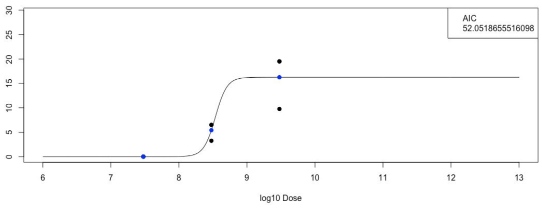

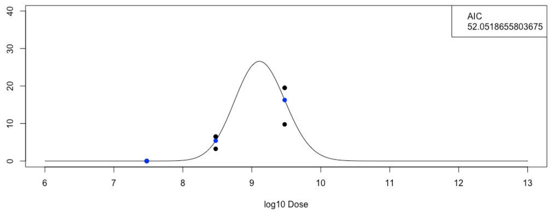

Response Type: CD8

Vector Species: B

Host Species: Mouse

Route of Administration: IM

Paper 1201: Impact of Recombinant Adenovirus Serotype 35 Priming versus Boosting of a Plasmodium falciparum Protein: Characterization of T- and B-Cell Responses to Liver-Stage Antigen 1

Day 14

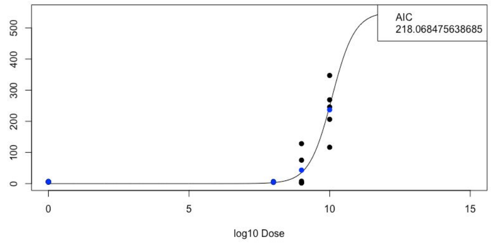

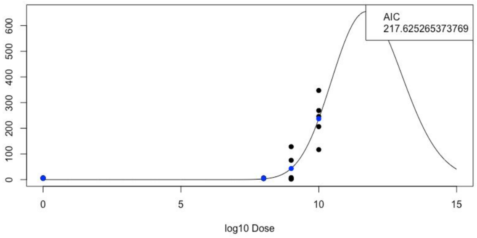

Paper 1269: Increased immunogenicity of recombinant Ad35-based malaria vaccine through formulation with aluminium phosphate adjuvant

Day 56

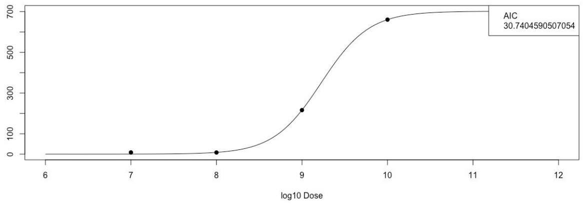

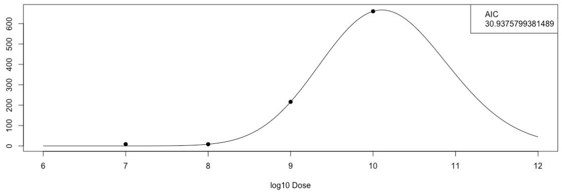

Paper 1492: Immunogenicity and Protection of a Recombinant Human Adenovirus Serotype 35-Based Malaria Vaccine against Plasmodium yoelii in Mice

Day 14

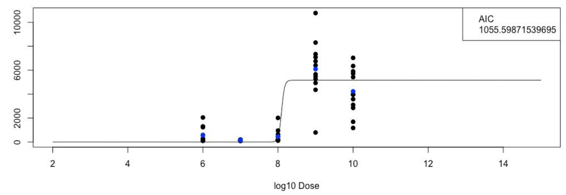

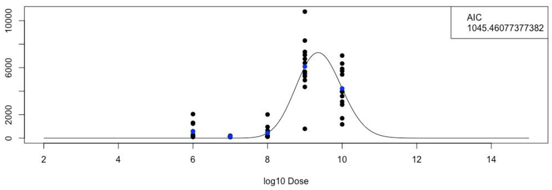

Route of Administration: SQ

Paper 555: Comparative analysis of the magnitude, quality, phenotype, and protective capacity of simian immunodeficiency virus gag-specific CD8+ T cells following human-, simian-, and chimpanzee-derived recombinant adenoviral vector immunization

rAd35

Day 14 - Tetramer Staining

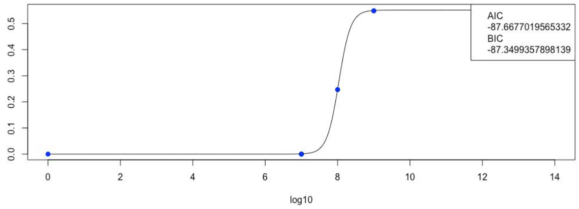

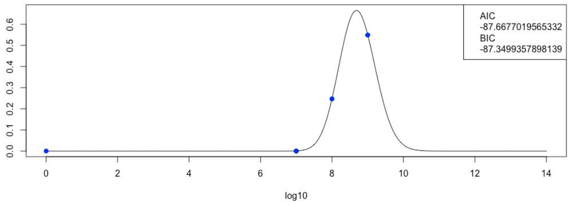

Day 21 - Tetramer Staining

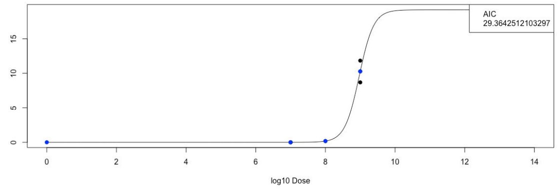

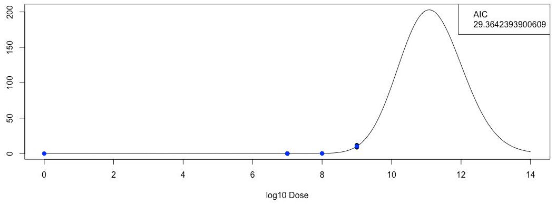

Day 28 - Tetramer Staining

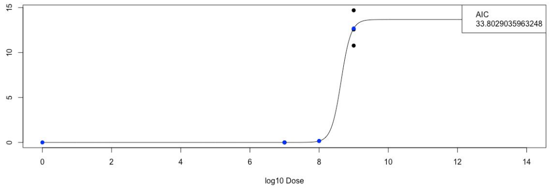

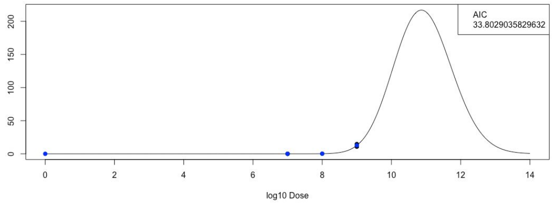

Day 35 - Tetramer Staining

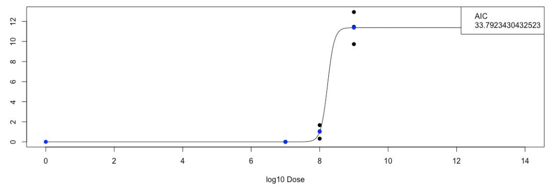

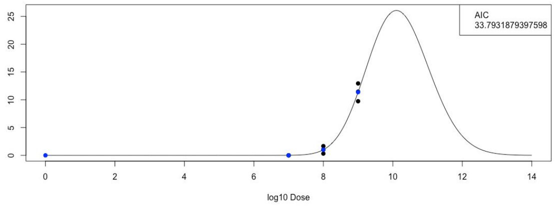

Day 70 - Tetramer Staining

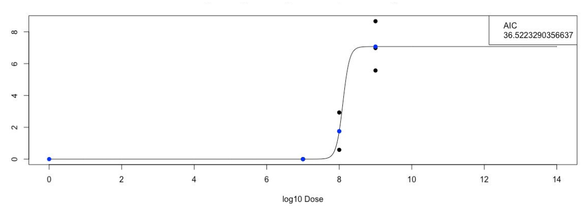

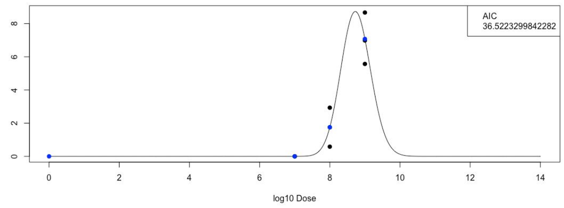

Day 23 - Cytokine Staining

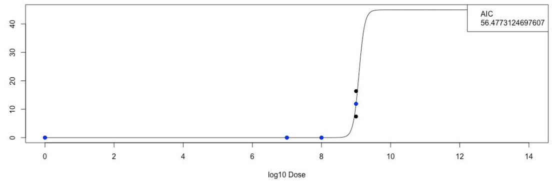

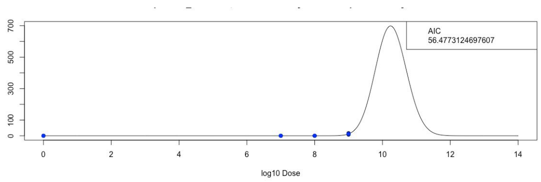

Day 70 -Cytokine Staining

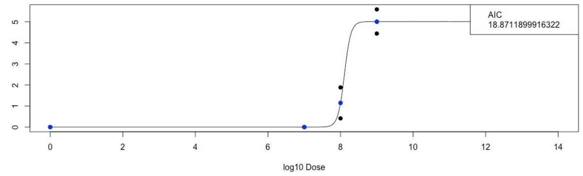

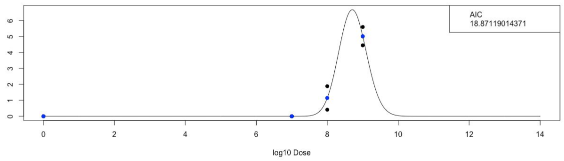

Host Species: Human

Route of Administration: IM

Paper 309: The novel tuberculosis vaccine, AERAS-402, is safe in healthy infants previously vaccinated with BCG, and induces dose-dependent CD4 and CD8T cell responses

Day 28 - Ag85A/b

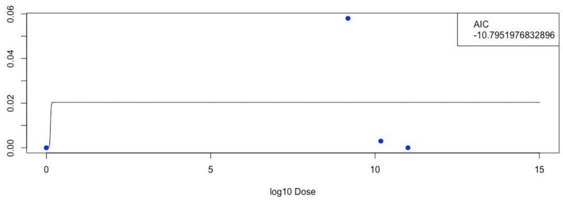

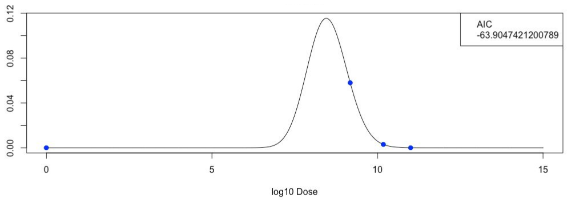

Day 28 - Tb.104

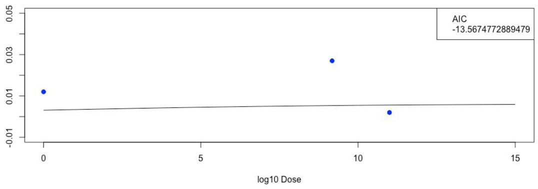

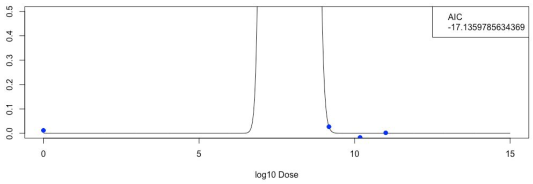

Vector Species: C

Host Species: Mouse

Route of Administration: IM

Paper 461: Beta-defensin 2 enhances immunogenicity and protection of an adenovirus-based H5N1 influenza vaccine at an early time

Day 7 HA-518

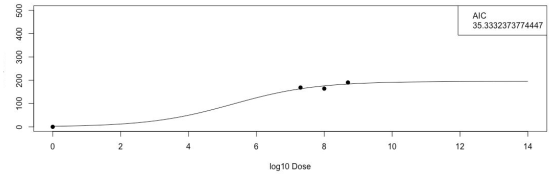

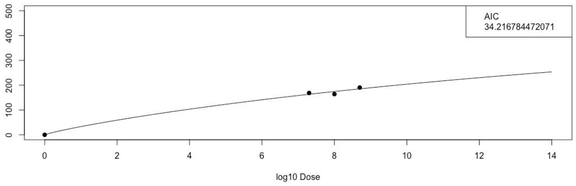

Day 14 HA-518

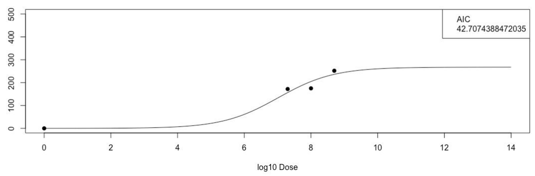

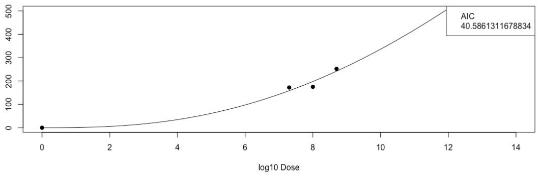

Day 7 NP-147

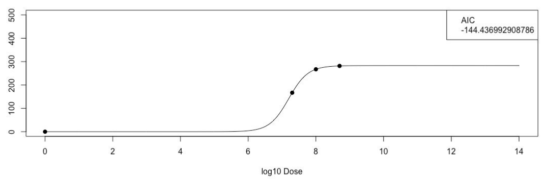

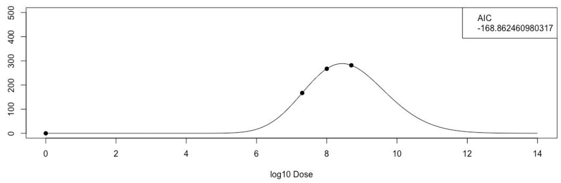

Day 14 NP-147

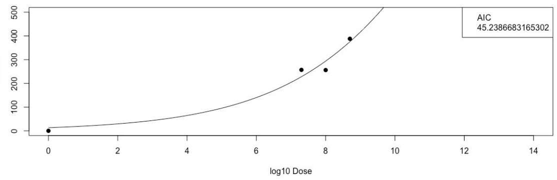

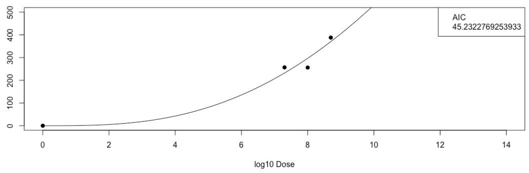

Paper 1039 Enhanced protection against Ebola virus mediated by an improved adenovirus-based vaccine

Day 8 - Ad-CAGoptZGP

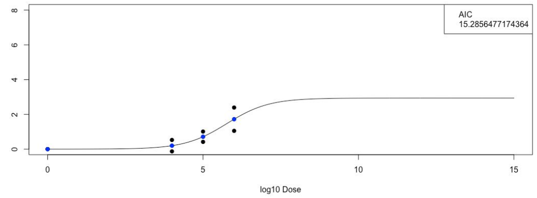

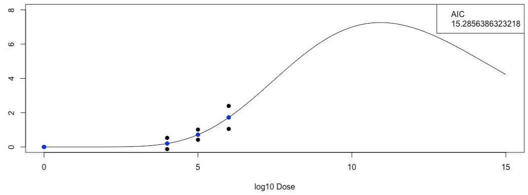

Day 8 - Ad-AdCMVZGP

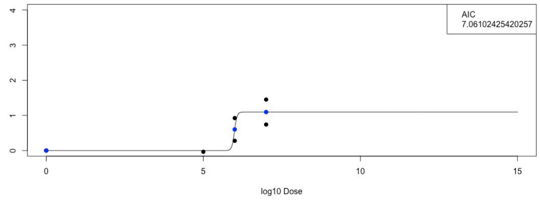

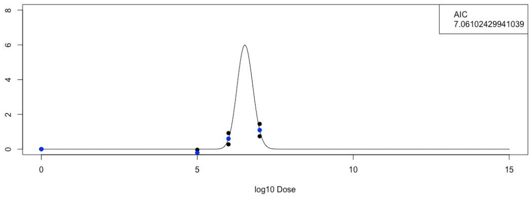

Paper 1492: Immunogenicity and Protection of a Recombinant Human Adenovirus Serotype 35-Based Malaria Vaccine against Plasmodium yoelii in Mice

Day 14

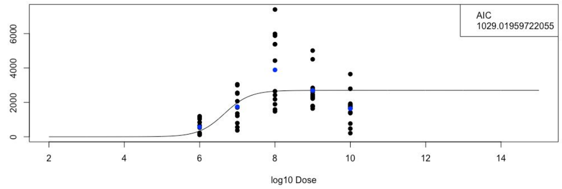

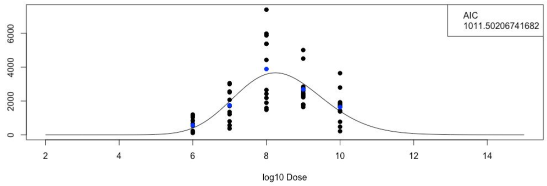

Paper 3018: Inhibitory receptor expression on memory CD8 T cells following Ad vector immunization

Day 7

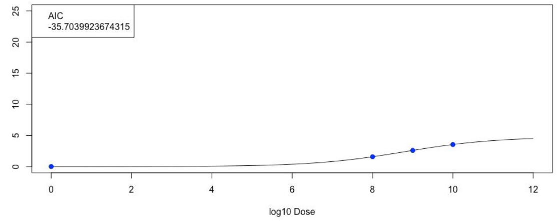

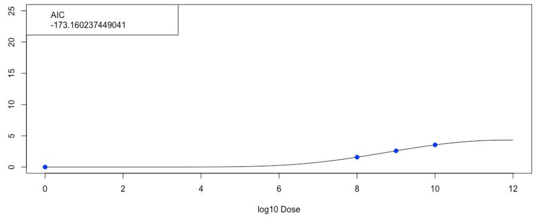

Day 15

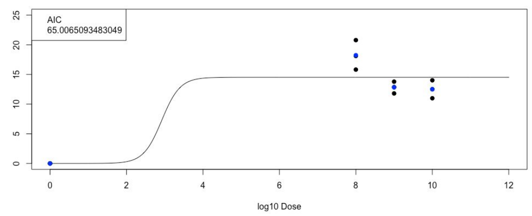

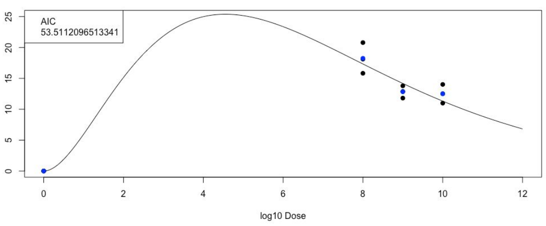

Day 30

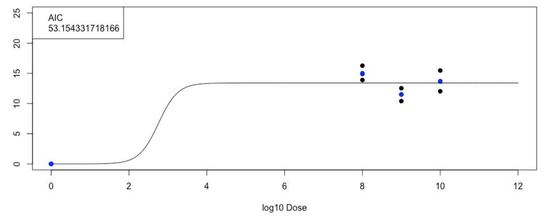

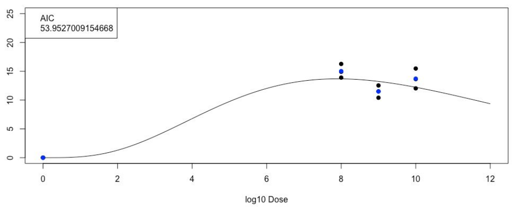

Route of Administration: SQ

Paper 555: Comparative analysis of the magnitude, quality, phenotype, and protective capacity of simian immunodeficiency virus gag-specific CD8+ T cells following human-, simian-, and chimpanzee-derived recombinant adenoviral vector immunization

rAd5

Day 14 - Tetramer Staining

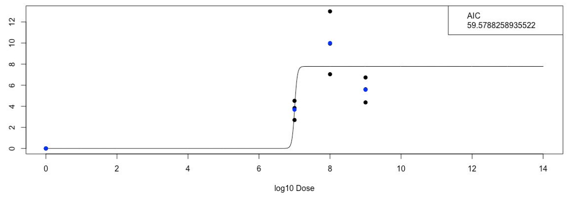

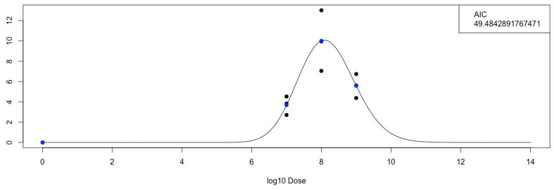

Day 21 - Tetramer Staining

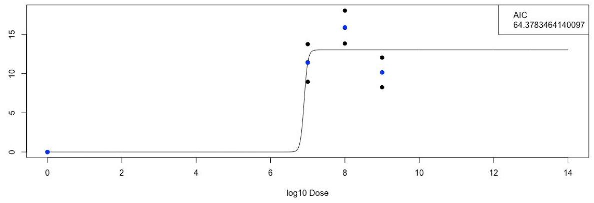

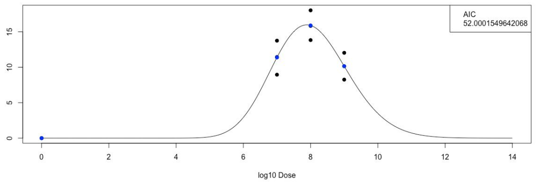

Day 28 - Tetramer Staining

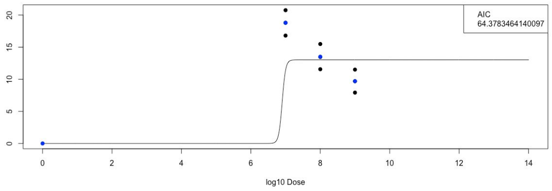

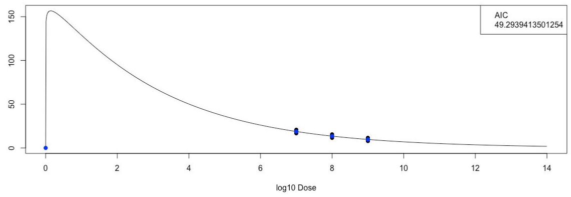

Day 35 - Tetramer Staining

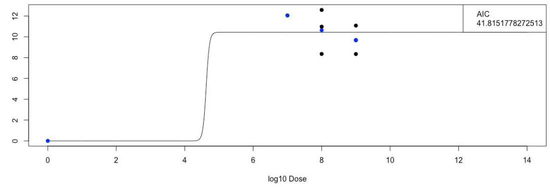

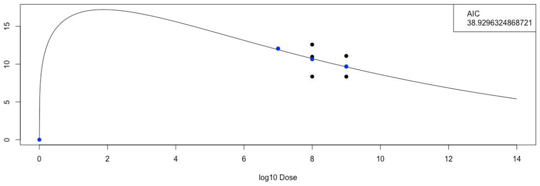

Day 70 - Tetramer Staining

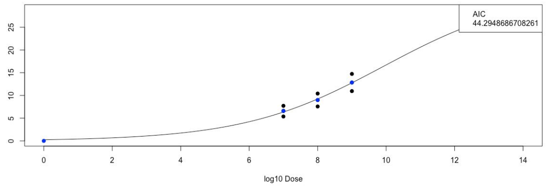

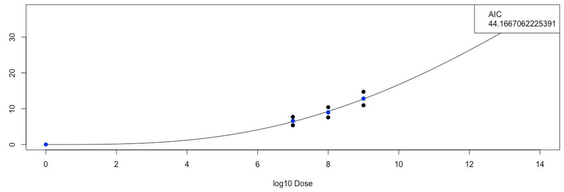

Day 23 - Cytokine Staining

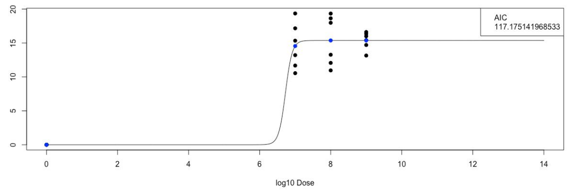

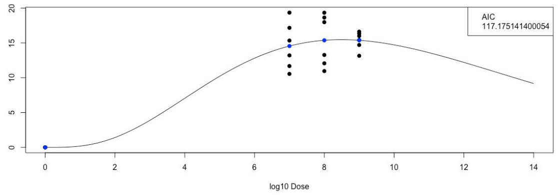

Day 70 -Cytokine Staining

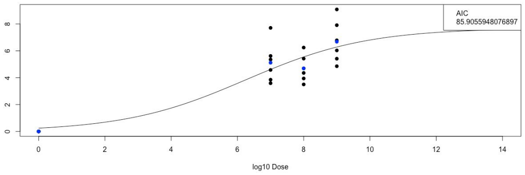

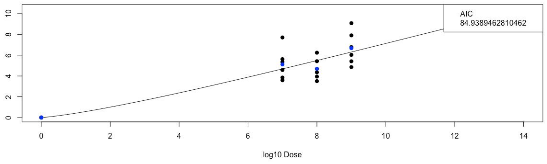

ChAd3

Day 14 - Tetramer Staining

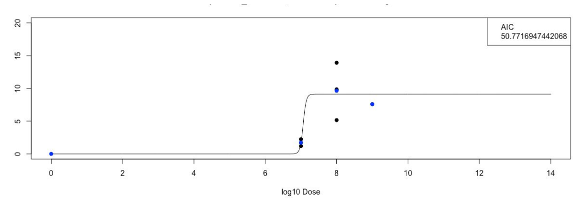

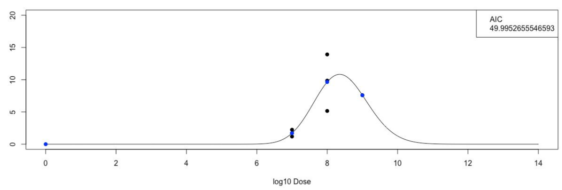

Day 21 - Tetramer Staining

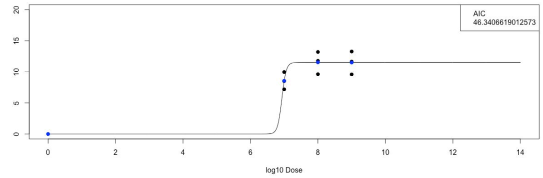

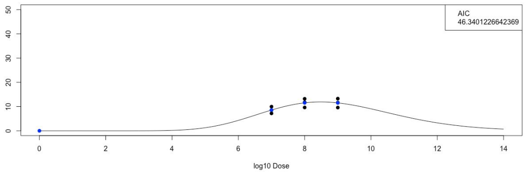

Day 28 - Tetramer Staining

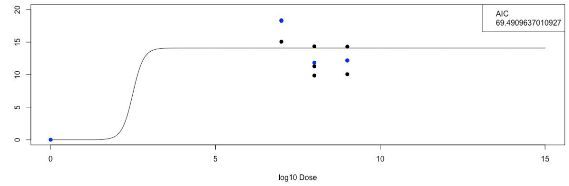

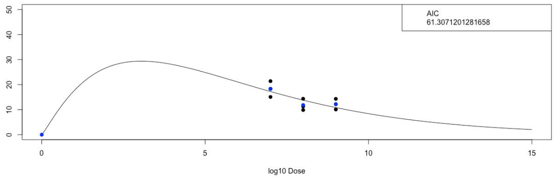

Day 35 - Tetramer Staining

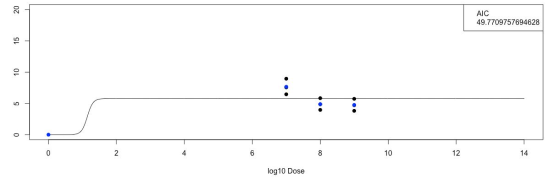

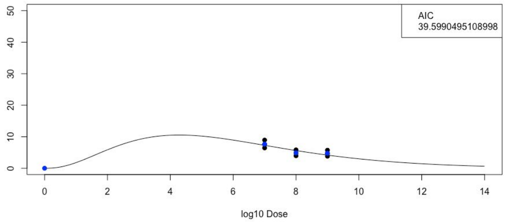

Day 70 - Tetramer Staining

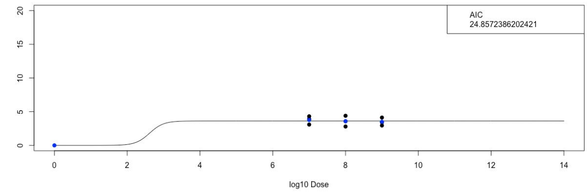

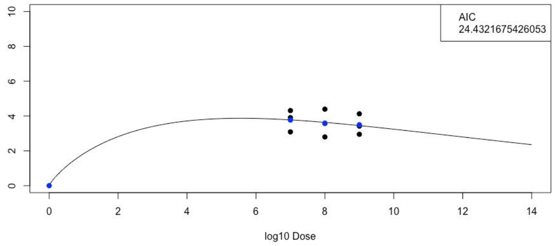

Day 23 - Cytokine Staining

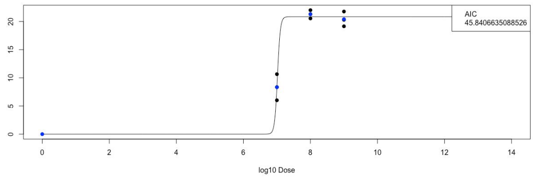

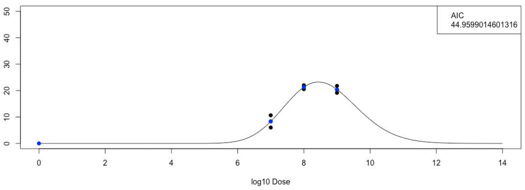

Day 70 -Cytokine Staining

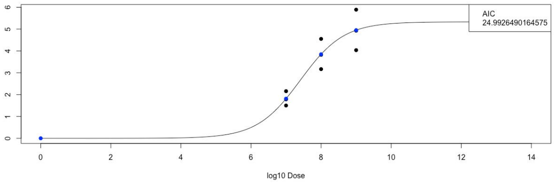

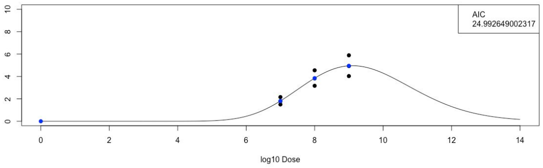

Paper 2980: Early life vaccination: Generation of adult-quality memory CD8+ T cells in infant mice using non-replicating adenoviral vectors

Day 60

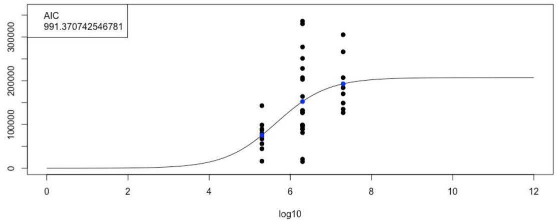

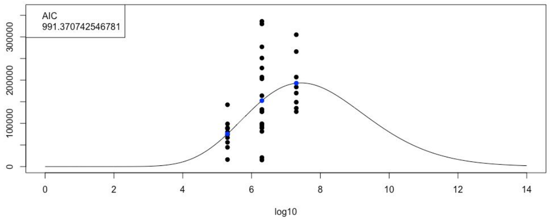

Host Species: Monkey

Route of Administration: IM

Paper 1474: A novel adenovirus type 6 (Ad6)-based hepatitis C virus vector that overcomes preexisting anti-ad5 immunity and induces potent and broad cellular immune responses in rhesus macaques

Day 8 - Ad5

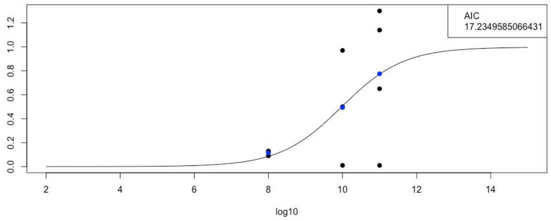

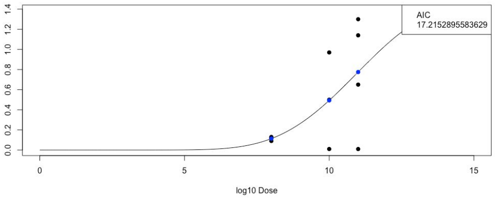

Day 8 - Ad6

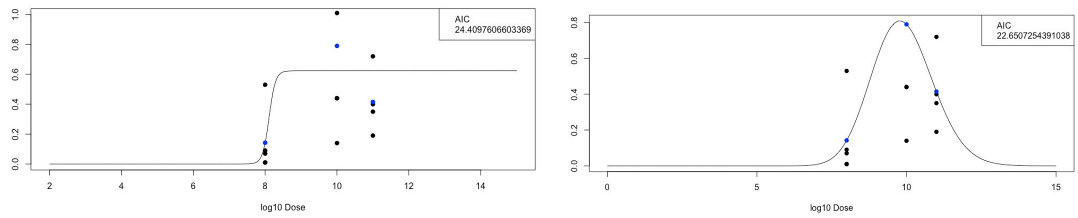

Vector Species: D

Host Species: Mouse

Route of Administration: IM

Paper 924 TLR4 Ligands Augment Antigen-Specific CD8+ T Lymphocyte Responses Elicited by a Viral Vaccine Vector

Day 14

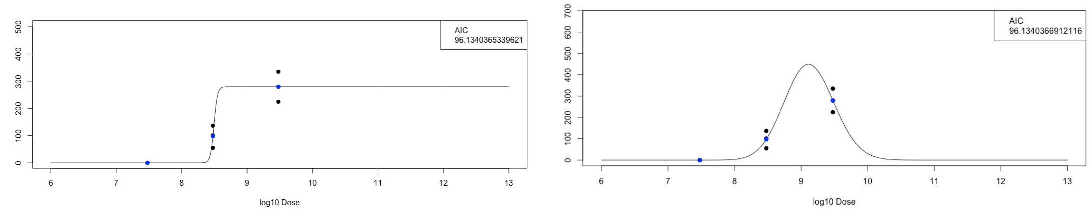

Route of Administration: SQ

Paper 555: Comparative analysis of the magnitude, quality, phenotype, and protective capacity of simian immunodeficiency virus gag-specific CD8+ T cells following human-, simian-, and chimpanzee-derived recombinant adenoviral vector immunization

rAd28
Day 14 - Tetramer Staining

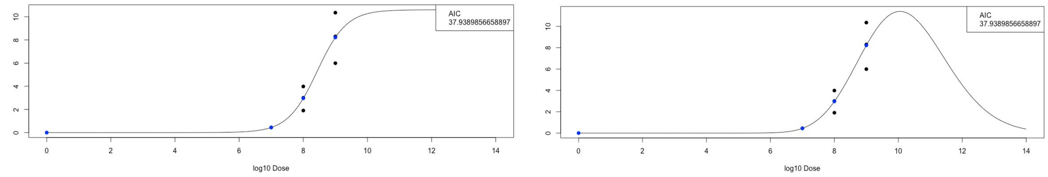

Day 21 - Tetramer Staining

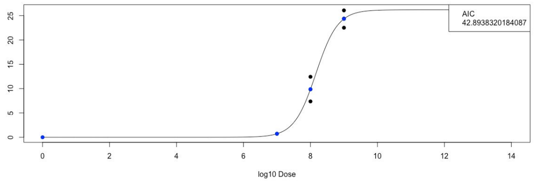

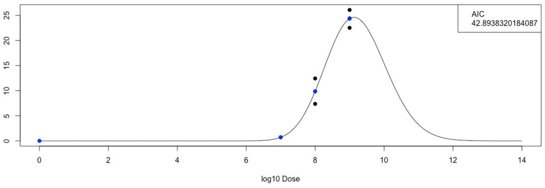

Day 28 - Tetramer Staining

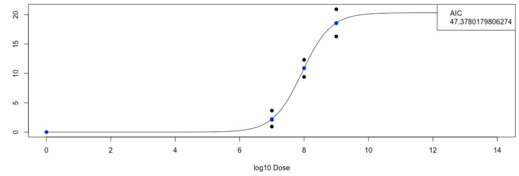

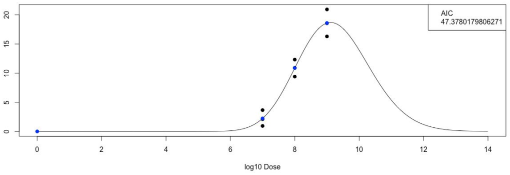

Day 35 - Tetramer Staining

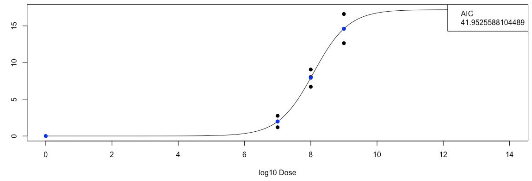

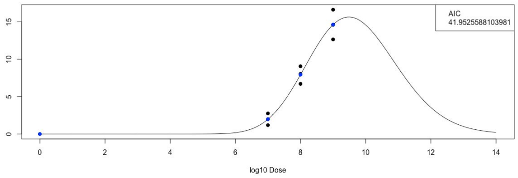

Day 70 - Tetramer Staining

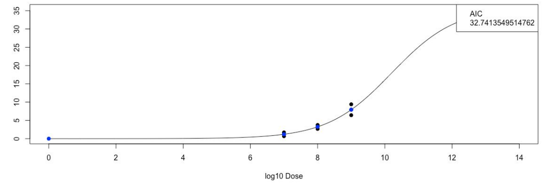

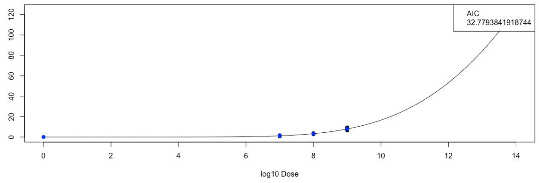

Day 23 - Cytokine Staining

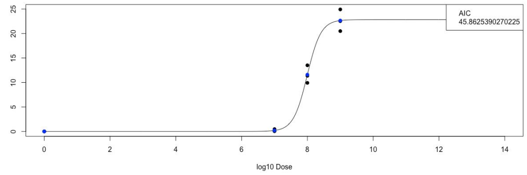

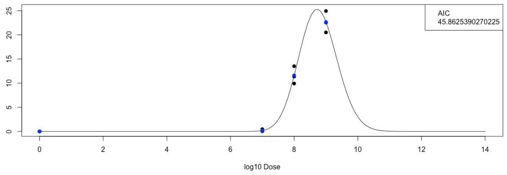

Day 70 -Cytokine Staining

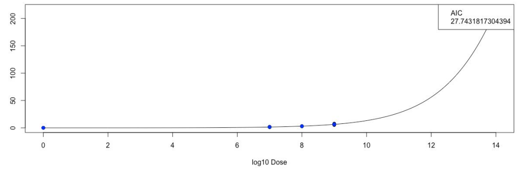

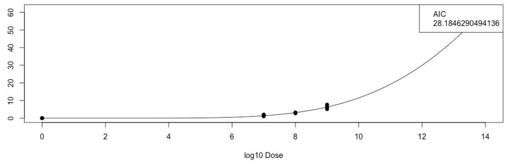

Vector Species: E

Host Species: Mouse

Route of Administration: IM

Paper 2919: A prime-boost immunization regimen based on a Simian Adenovirus 36 vectored multi-stage malaria vaccine induces protective immunity in mice.

Day 10

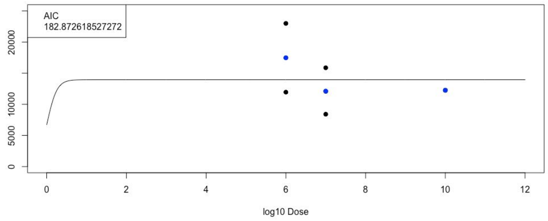

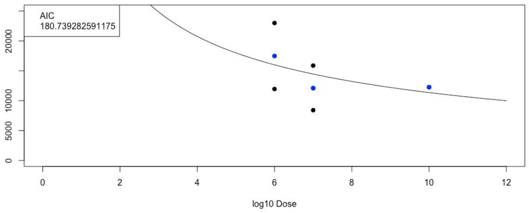

Day 20

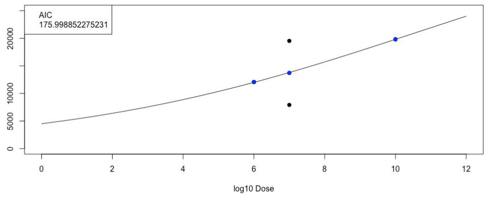

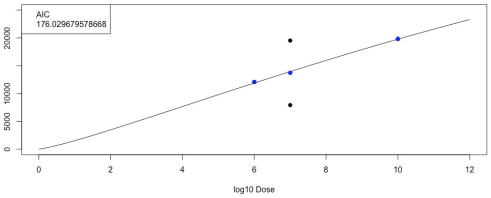

Route of Administration: SQ

Paper 555: Comparative analysis of the magnitude, quality, phenotype, and protective capacity of simian immunodeficiency virus gag-specific CD8+ T cells following human-, simian-, and chimpanzee-derived recombinant adenoviral vector immunization

ChAd63

Day 14 - Tetramer Staining

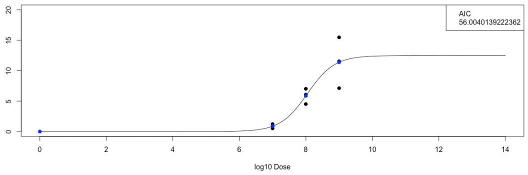

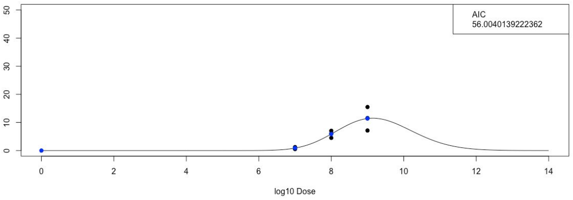

Day 21 - Tetramer Staining

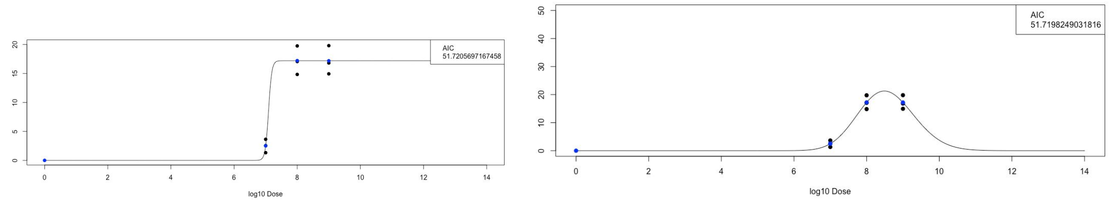

Day 28 - Tetramer Staining

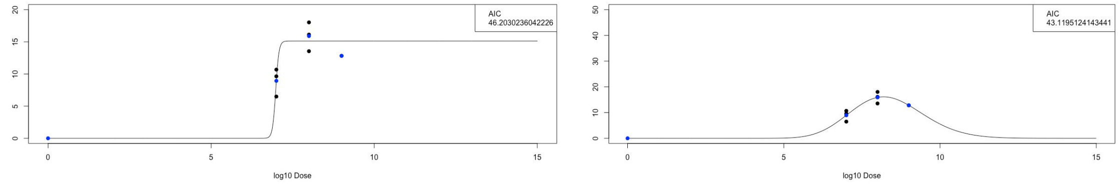

Day 35 - Tetramer Staining

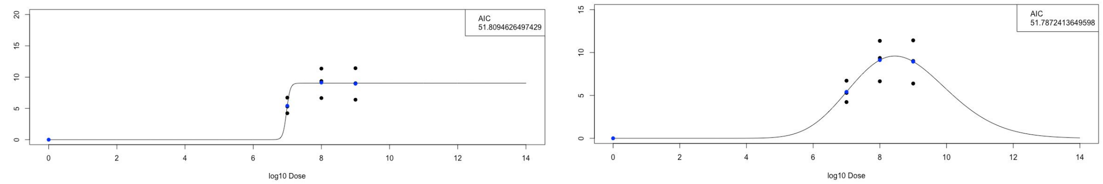

Day 70 - Tetramer Staining

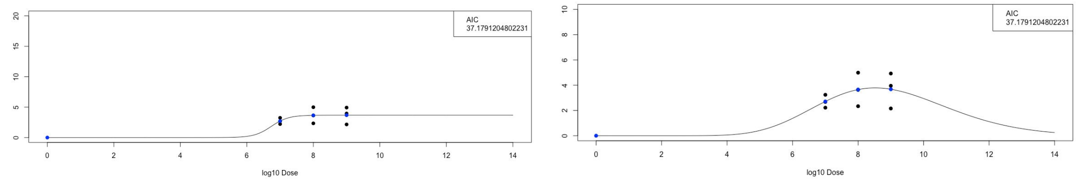

Day 23 - Cytokine Staining

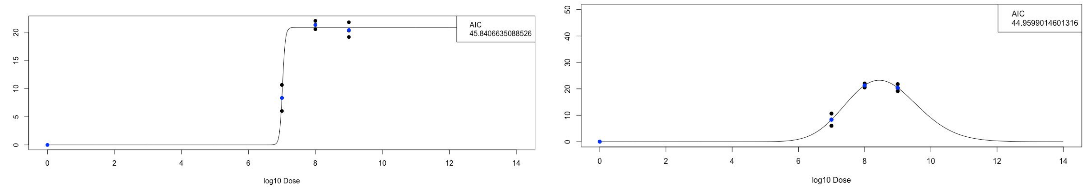

Day 70 -Cytokine Staining

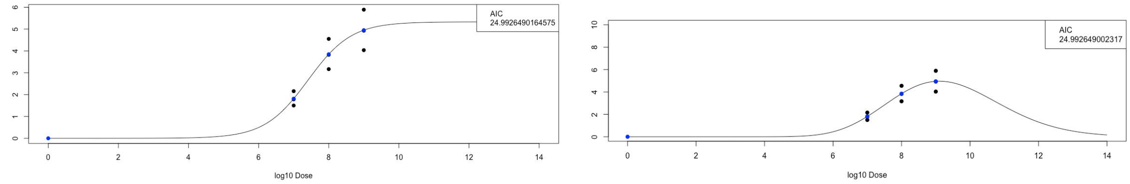

Vector Species: G

Host Species: Mouse

Route of Administration: SQ

Paper 555: Comparative analysis of the magnitude, quality, phenotype, and protective capacity of simian immunodeficiency virus gag-specific CD8+ T cells following human-, simian-, and chimpanzee-derived recombinant adenoviral vector immunization

sAd11

Day 14 - Tetramer Staining

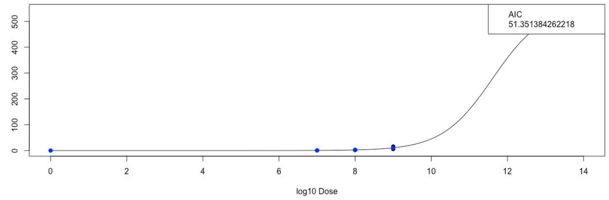

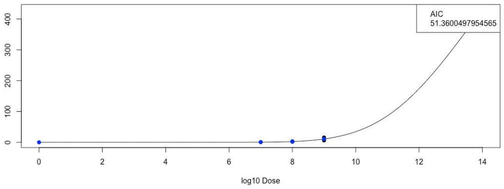

Day 21 - Tetramer Staining

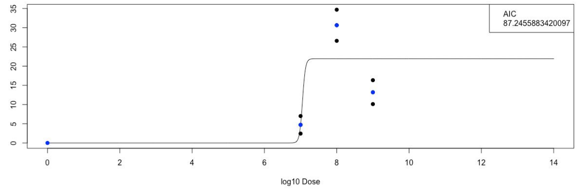

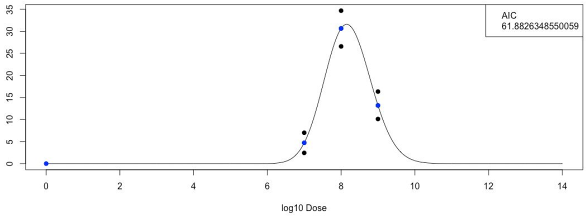

Day 28 - Tetramer Staining

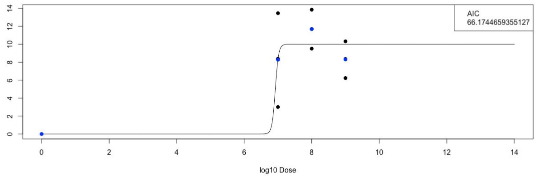

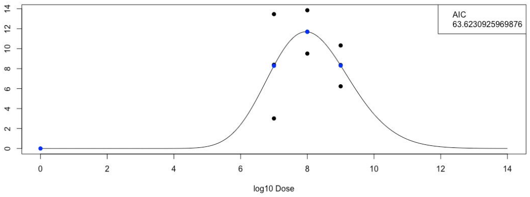

Day 35 - Tetramer Staining

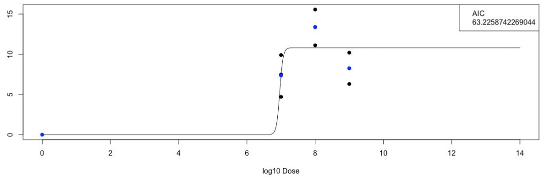

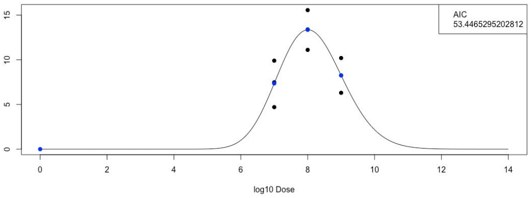

Day 70 - Tetramer Staining

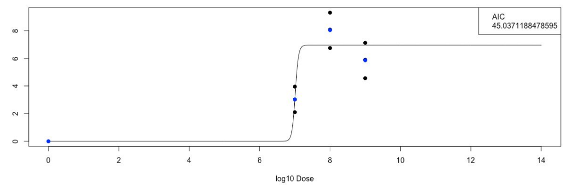

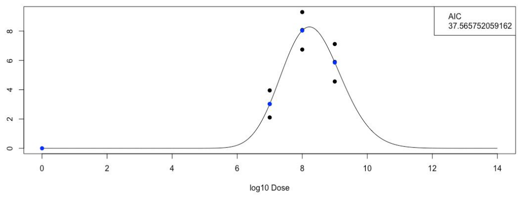

Day 23 - Cytokine Staining

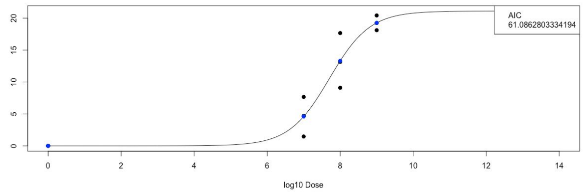

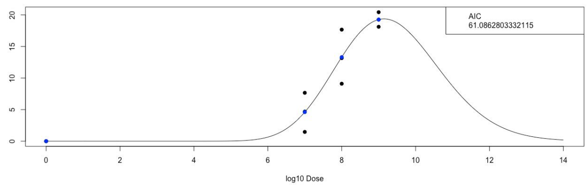

Day 70 -Cytokine Staining

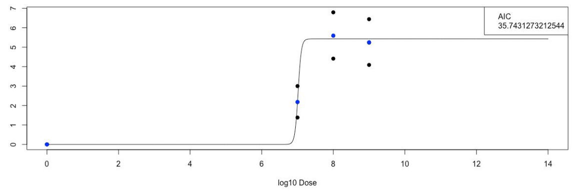

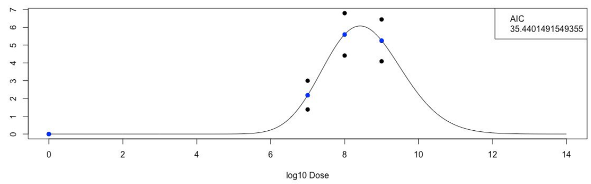

Vector Species: None

Host Species: Mouse

Route of Administration: SQ

Paper 555: Comparative analysis of the magnitude, quality, phenotype, and protective capacity of simian immunodeficiency virus gag-specific CD8+ T cells following human-, simian-, and chimpanzee-derived recombinant adenoviral vector immunization

sAd16

Day 14 - Tetramer Staining

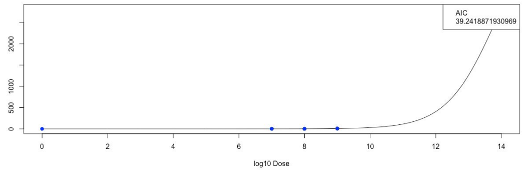

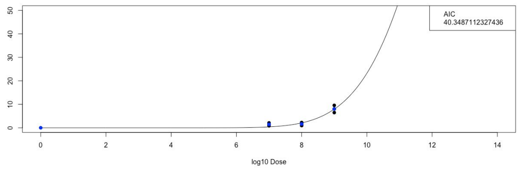

Day 21 - Tetramer Staining

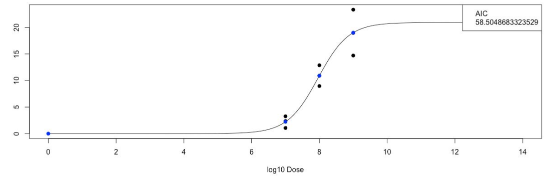

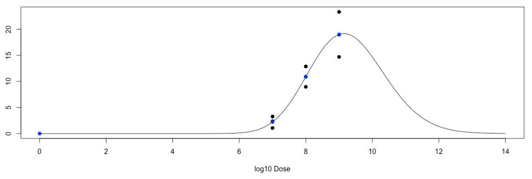

Day 28 - Tetramer Staining

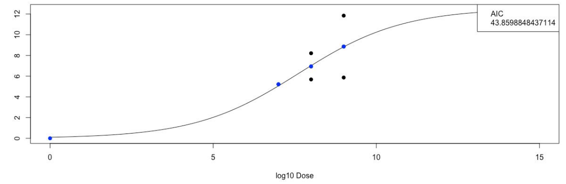

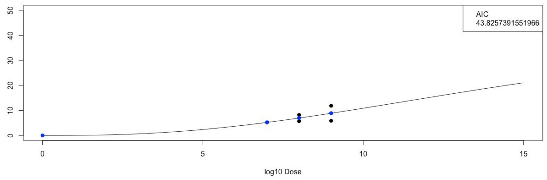

Day 35 - Tetramer Staining

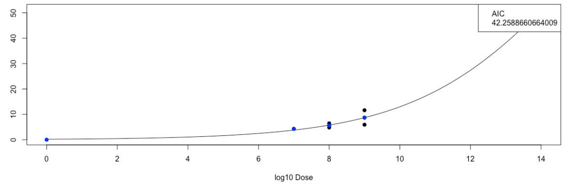

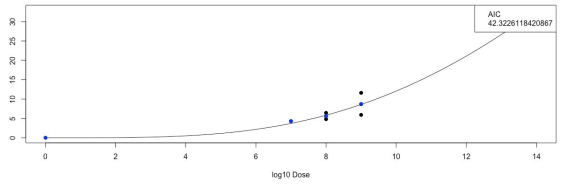

Day 70 - Tetramer Staining

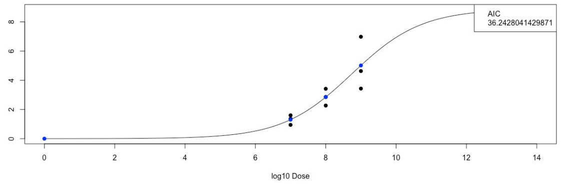

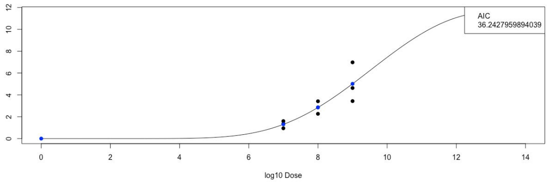

Day 23 - Cytokine Staining

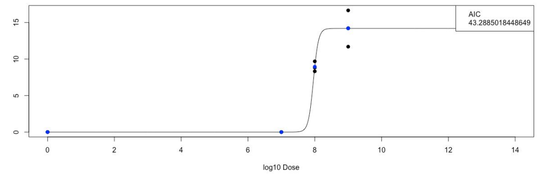

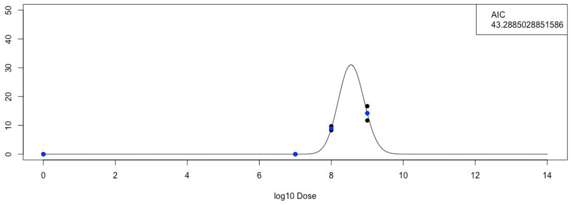

Day 70 -Cytokine Staining

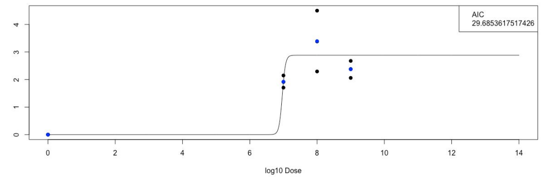

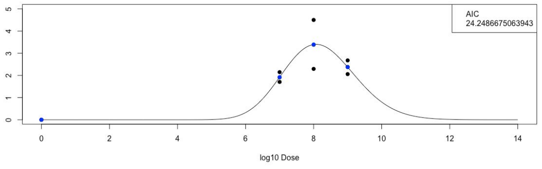

Response Type: CD4+ IFN+%

Vector Species: B

Host Species: Human

Route of Administration: IM

Paper 309: The novel tuberculosis vaccine, AERAS-402, is safe in healthy infants previously vaccinated with BCG, and induces dose-dependent CD4 and CD8T cell responses

Day 28

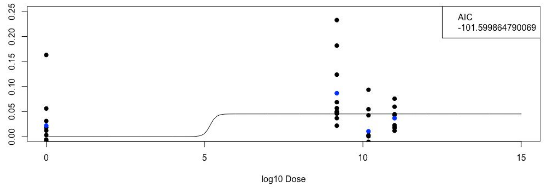

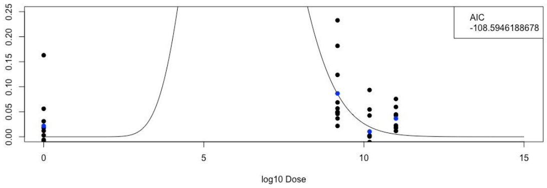

Paper 441: A phase 1b randomized, controlled, double-blinded dosage-escalation trial to evaluate the safety, reactogenicity and immunogenicity of an adenovirus type 35 based circumsporozoite malaria vaccine in Burkinabe healthy adults 18 to 45 years of age

Day 28

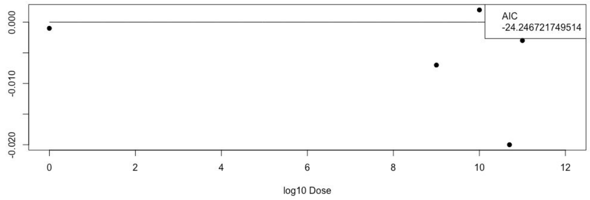

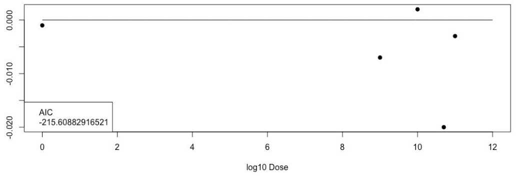

Response Type: CD8+ IFN+%

Vector Species: B

Host Species: Human

Route of Administration: IM

Paper 309: The novel tuberculosis vaccine, AERAS-402, is safe in healthy infants previously vaccinated with BCG, and induces dose-dependent CD4 and CD8T cell responses

Day 28

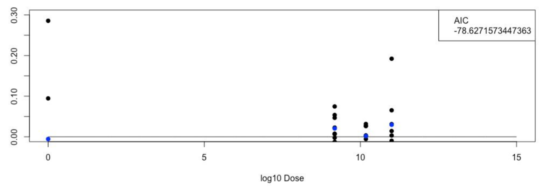

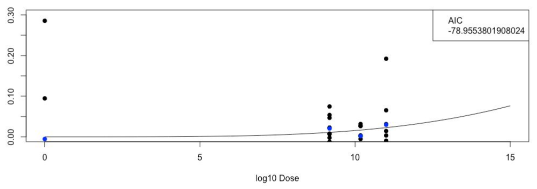

Paper 441: A phase 1b randomized, controlled, double-blinded dosage-escalation trial to evaluate the safety, reactogenicity and immunogenicity of an adenovirus type 35 based circumsporozoite malaria vaccine in Burkinabe healthy adults 18 to 45 years of age

Day 28

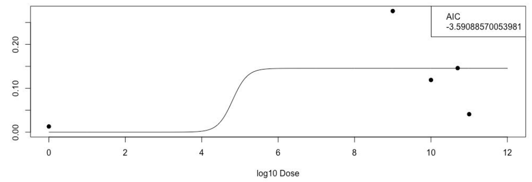

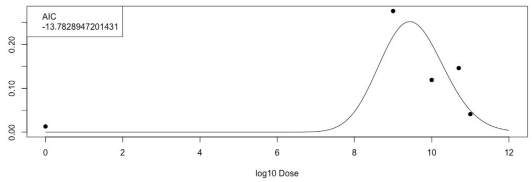

Vector Species: E

Host Species: Mouse

Route of Administration: IM

Paper 1801: Induction of CD8+ T cells to an HIV-1 antigen through a prime boost regimen with heterologous E1-deleted adenoviral vaccine carriers

Day 10

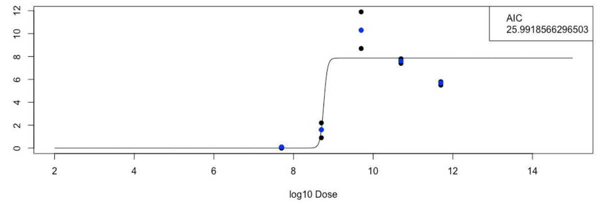

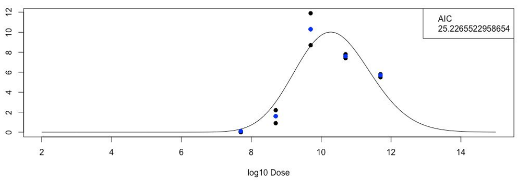

Response Type: CD4+ TNF+%

Vector Species: B

Host Species: Human

Route of Administration: IM

Paper 309: The novel tuberculosis vaccine, AERAS-402, is safe in healthy infants previously vaccinated with BCG, and induces dose-dependent CD4 and CD8T cell responses

Day 28

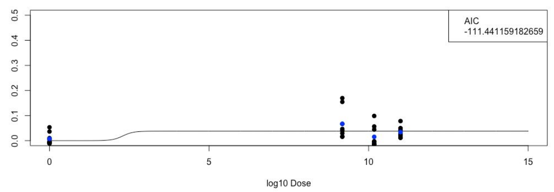

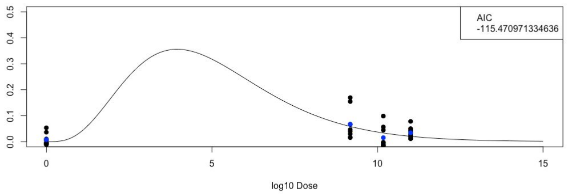

Paper 441: A phase 1b randomized, controlled, double-blinded dosage-escalation trial to evaluate the safety, reactogenicity and immunogenicity of an adenovirus type 35 based circumsporozoite malaria vaccine in Burkinabe healthy adults 18 to 45 years of age

Day 28

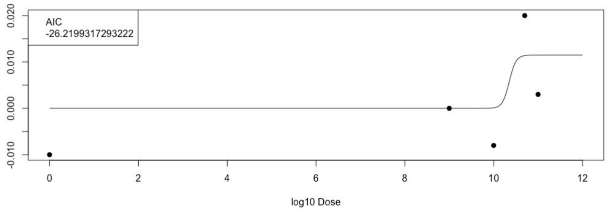

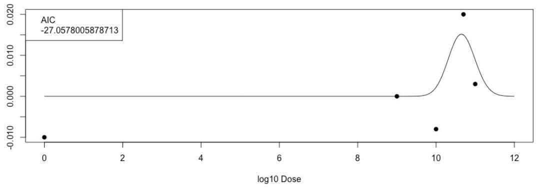

Response Type: CD8+ TNF+%

Vector Species: B

Host Species: Human

Route of Administration: IM

Paper 441: A phase 1b randomized, controlled, double-blinded dosage-escalation trial to evaluate the safety, reactogenicity and immunogenicity of an adenovirus type 35 based circumsporozoite malaria vaccine in Burkinabe healthy adults 18 to 45 years of age

Day 28

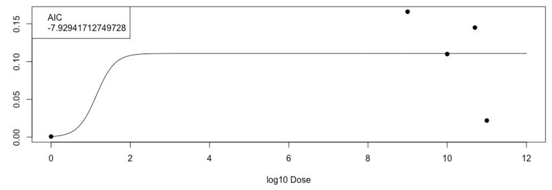

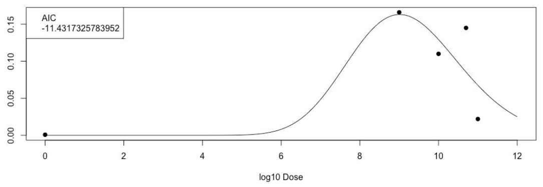

Response Type: CD4+ Il2+%

Vector Species: B

Host Species: Human

Route of Administration: IM

Paper 309: The novel tuberculosis vaccine, AERAS-402, is safe in healthy infants previously vaccinated with BCG, and induces dose-dependent CD4 and CD8T cell responses

Day 28

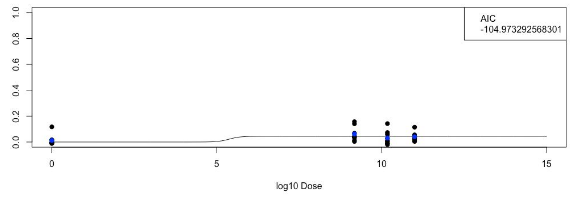

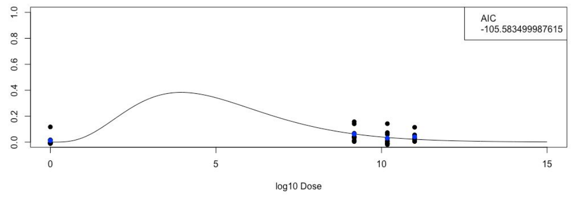

Paper 441: A phase 1b randomized, controlled, double-blinded dosage-escalation trial to evaluate the safety, reactogenicity and immunogenicity of an adenovirus type 35 based circumsporozoite malaria vaccine in Burkinabe healthy adults 18 to 45 years of age

Day 28

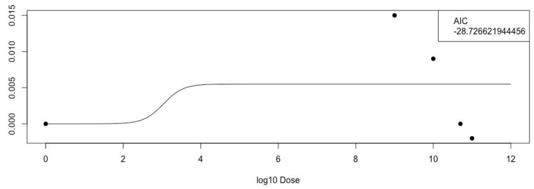

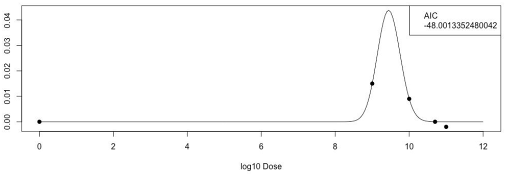

Response Type: CD8+ Il2+%

Vector Species: B

Host Species: Human

Route of Administration: IM

Paper 441: A phase 1b randomized, controlled, double-blinded dosage-escalation trial to evaluate the safety, reactogenicity and immunogenicity of an adenovirus type 35 based circumsporozoite malaria vaccine in Burkinabe healthy adults 18 to 45 years of age

Day 28

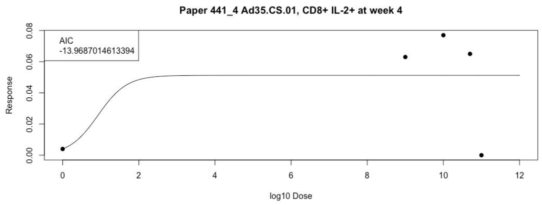

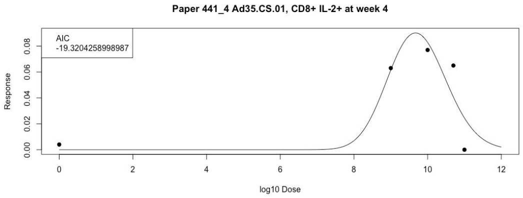

Response Type: CD4+ Il17+%

Vector Species: B

Host Species: Human

Route of Administration: IM

Paper 309: The novel tuberculosis vaccine, AERAS-402, is safe in healthy infants previously vaccinated with BCG, and induces dose-dependent CD4 and CD8T cell responses

Day 28

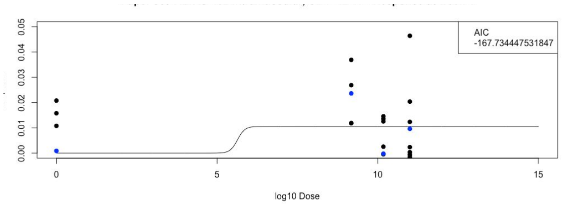

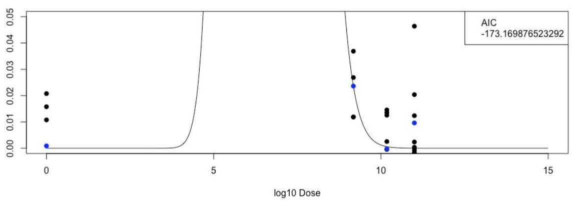

Response Type: Virus Neutralisation Titre

Vector Species: B

Host Species: Mouse

Route of Administration: IM

Paper 1269: Increased immunogenicity of recombinant Ad35-based malaria vaccine through formulation with aluminium phosphate adjuvant

Day 56

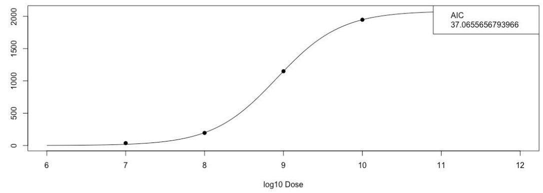

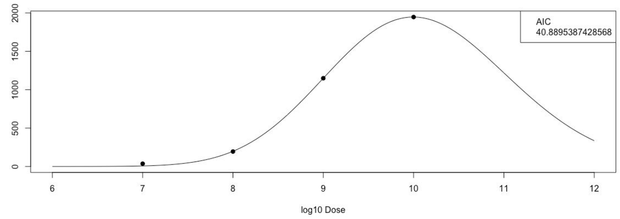

Route of Administration: SQ

Paper 2030: Novel, Chimpanzee Serotype 68-Based Adenoviral Vaccine Carrier for Induction of Antibodies to a Transgene Product

Day 14

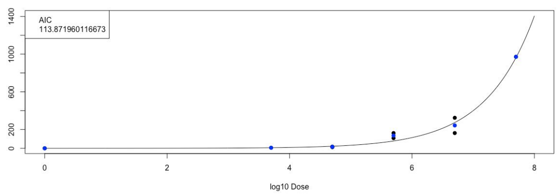

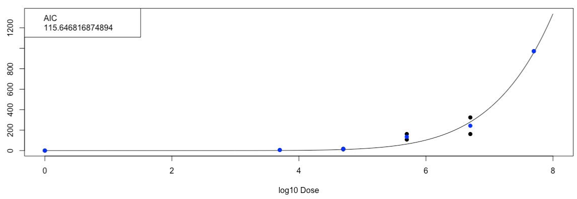

Vector Species: C

Host Species: Mouse

Route of Administration: IM

Paper 461: Beta-defensin 2 enhances immunogenicity and protection of an adenovirus-based H5N1 influenza vaccine at an early time

Day 7

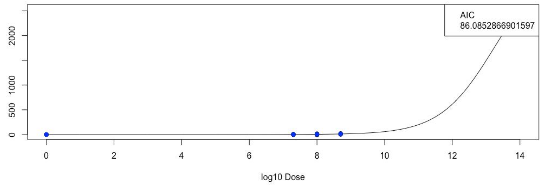

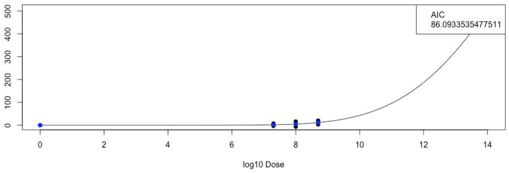

Day 14

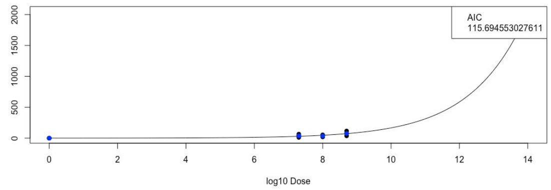

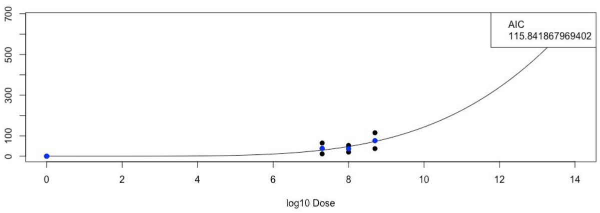

Paper 574: Recombinant adenovirus expressing type Asia1 foot-and-mouth disease virus capsid proteins induces protective immunity against homologous virus challenge in mice

Day 7

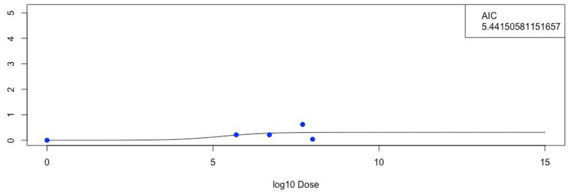

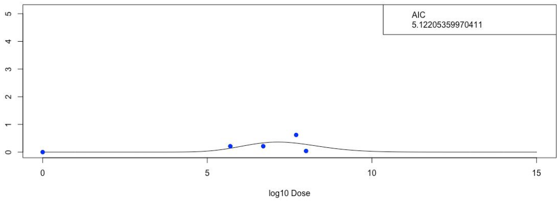

Day 21

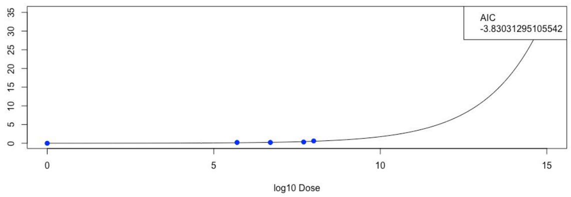

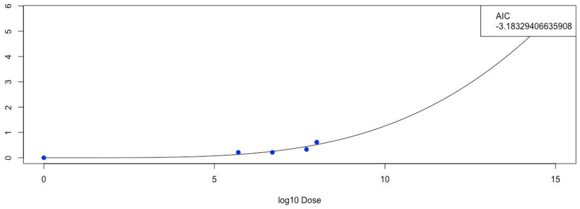

Day 35

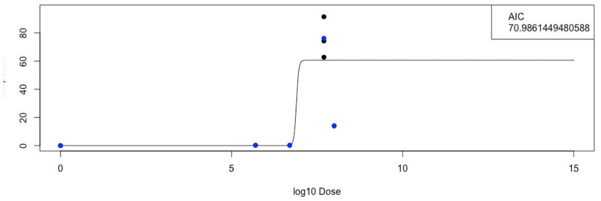

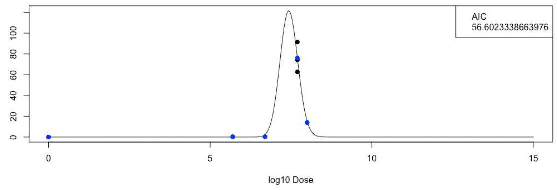

Day 49

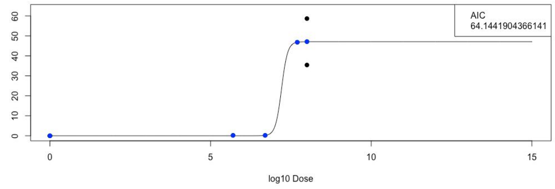

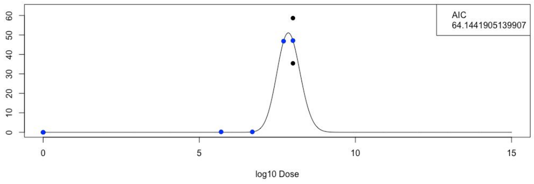

Day 63

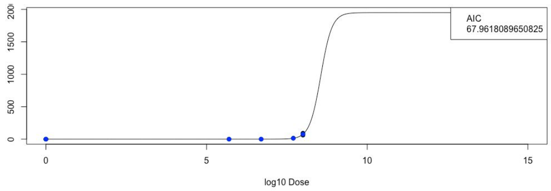

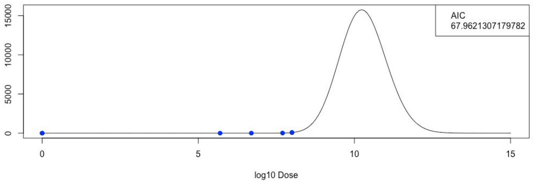

Day 77

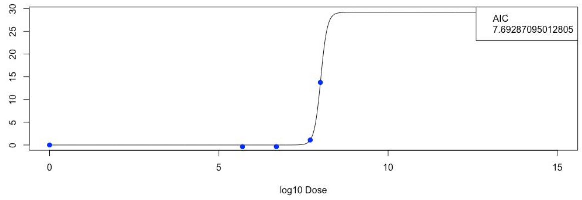

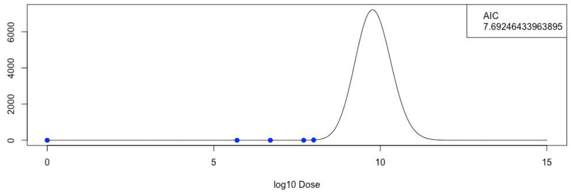

Day 119

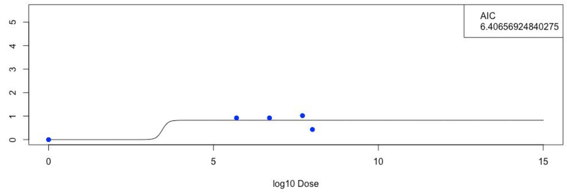

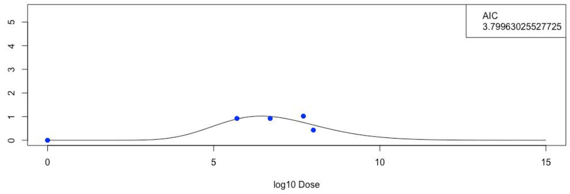

Day 161

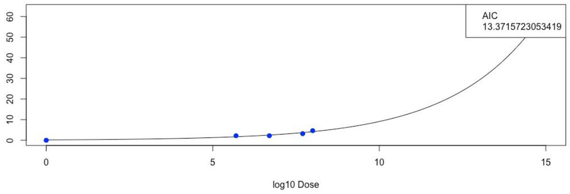

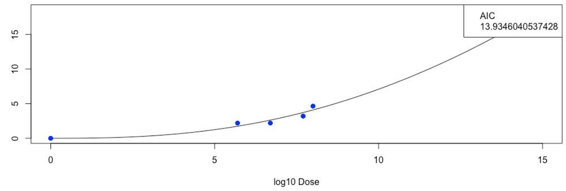

Paper 669: Impact of preexisting adenovirus vector immunity on immunogenicity and protection conferred with an adenovirus-based H5N1 influenza vaccine

Day 28

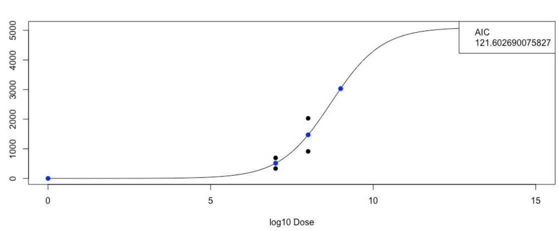

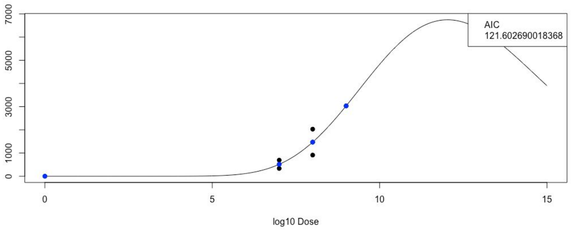

Paper 1039 Enhanced protection against Ebola virus mediated by an improved adenovirus-based vaccine

Day 8 - Ad-CAGoptZGP

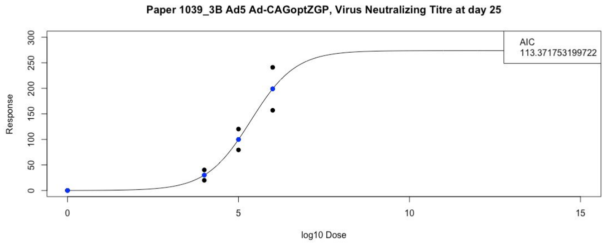

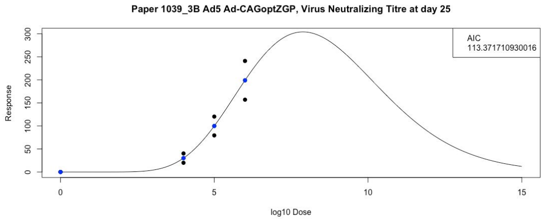

Route of Administration: SQ

Paper 2505: A replication-defective human adenovirus recombinant serves as a highly efficacious vaccine carrier

Day 10

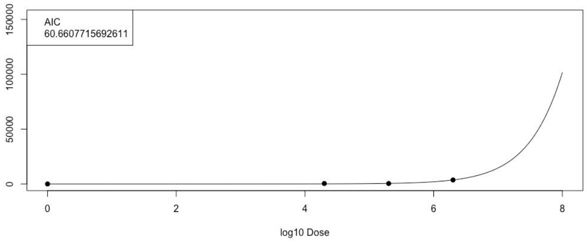

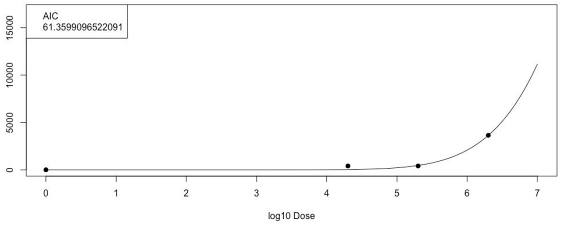

Day 14

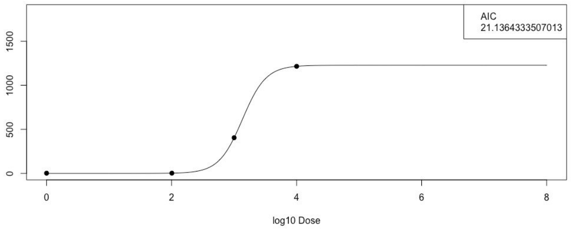

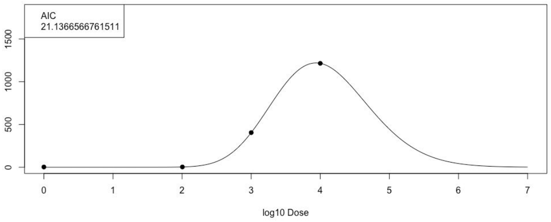

Host Species: Cattle

Route of Administration: IM

Paper 2841: Efficacy of an adenovirus-vectored foot-and-mouth disease virus serotype A subunit vaccine in cattle using a direct contact transmission model

Day 7

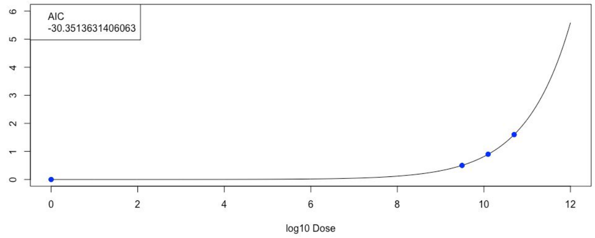

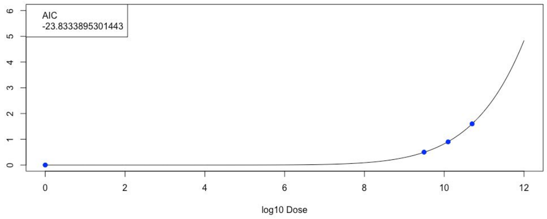

Vector Species: D

Host Species: Human

Route of Administration: IM

Paper 594: First-in-human evaluation of the safety and immunogenicity of a recombinant adenovirus serotype 26 HIV-1 Env vaccine (IPCAVD 001)

Day 14

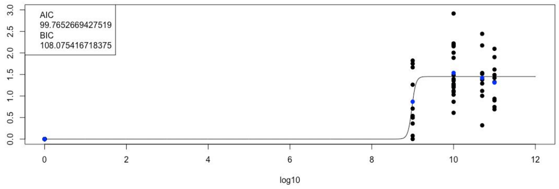

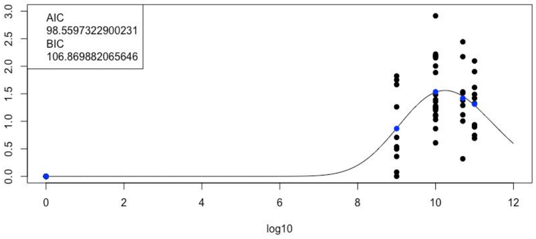

Host Species: Rat

Route of Administration: IM

Paper 594: Recombinant low-seroprevalent adenoviral vectors Ad26 and Ad35 expressing the respiratory syncytial virus (RSV) fusion protein induce protective immunity against RSV infection in cotton rats

Day 28

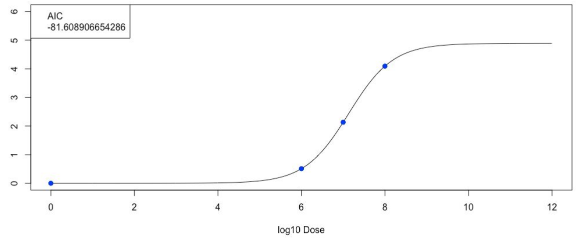

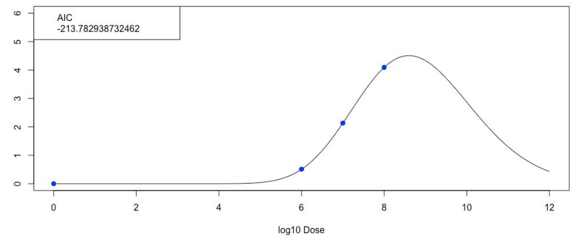

Vector Species: E

Host Species: Mouse

Route of Administration: SQ

Paper 2030: Novel, Chimpanzee Serotype 68-Based Adenoviral Vaccine Carrier for Induction of Antibodies to a Transgene Product

Day 14

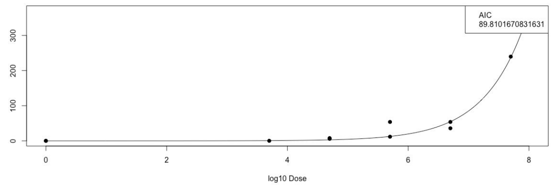

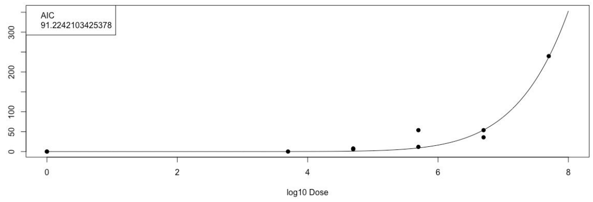

Host Species: Human

Route of Administration: IM

Paper 686: Clinical assessment of a recombinant simian adenovirus ChAd63: a potent new vaccine vector

Day 14

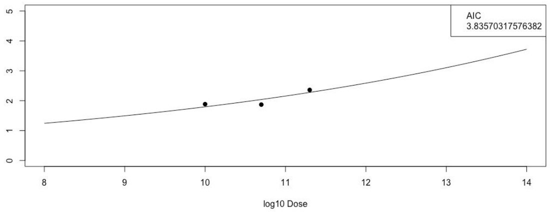

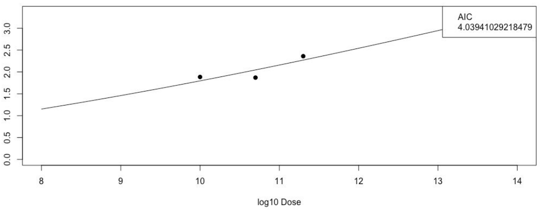

Day 21

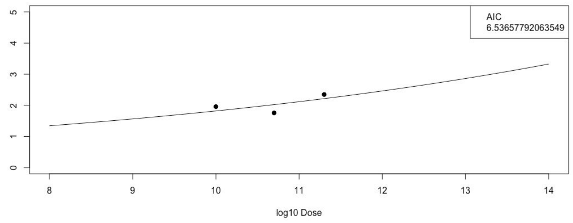

AIC
6.53657792063549
log10 Dose

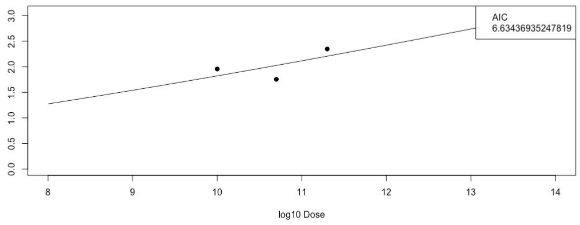

AIC
6.63436935247819
log10 Dose

Supplementary Tables

These tables summarise the datasets in Supplementary Figures S1 including the level of evidence towards either a peaking or saturating curve shape, determined by AIC difference. An ‘x’ is used to identify the level of evidence. There exists a table for each response type. Bold lines are used to show when the group the dataset belongs to changes. The left most column combined with the response type of the table gives the group. Each dataset also includes the paper number the dataset was gathered from and the days post inoculation that the responses were measured.

S1 Antibody

Antibodies									
Group<br>(Adenoviral Species/Host Species/Administration Route)	Paper	Days post inoculation	Absolute Peaking Evidence (<10)	Strong Peaking Evidence (10-6)	Slight Peaking Evidence (2-6)	No Evidence (<2)	Slight Saturating Evidence (2-6)	Strong Saturating Evidence (10-6)	Strong Saturating Evidence (<10)
B/Mouse/IM	578	28				x			
		28				x			
		28				x			
		28				x			
		28				x			
	1269	56				x			
	1492	14				x			
B/Human/IM	441	28				x			
	467	60			x				
	633	28	x						

C/Rabbit/IM	744	28				x			
C/Mouse/IM	461	7				x			
		14				x			
	574	7				x			
		21				x			
		35			x				
		49				x			
		63				x			
		77				x			
		91				x			
		119				x			
		147				x			
		161				x			
	1492	14				x			
	2531	10	x						
		10				x			
C/Mouse/SQ	936	35				x			
		35				x			
C/Human/IM	140	28				x			
	249	14				x			
		28				x			

		180				x			
C/Monkey/IM	1877	28						x	
		56							x
		84				x			
		112					x		
		140				x			
C/Rat/IM	2531	10				x			
		10	x						
D/Mouse/IM	578	28				x			
		28				x			
		28				x			
		28				x			
		28				x			
D/Human/IM	594	14				x			
E/Mouse/IM	1539	14				x			
		28				x			
		42				x			
		14				x			
		28				x			
		42				x			
	2919	20				x			

E/Human/IM	417	14				x			
		21				x			
Count			3	0	2	46	1	1	1

S2 T Cells

T Cells									
Group (Adenoviral Species/Host Species/Administration Route)	Paper	Days post inoculation	Absolute Peaking Evidence (<10)	Strong Peaking Evidence (10-6)	Slight Peaking Evidence (2-6)	No Evidence	Slight Saturating Evidence (2-6)	Strong Saturating Evidence (10-6)	Strong Saturating Evidence (<10)
B/Mouse/IM	578	28				x			
		28				x			
		28				x			
		28	x						
		28				x			
	1269	56				x			
B/Human/IM	441	28		x					
	633	14				x			
		28				x			
C/Mouse/IM	2916	25				x			
		25				x			
		25				x			
		25					x		
		25					x		
		25					x		
C/Monkey/IM	1474	8				x			
		24				x			
		8				x			
		24				x			
	1877	28				x			
		168				x			
D/Mouse/IM	578	28				x			
		28				x			
		28				x			
		28						x	

		28				x			
	924	14				x			
D/Human/IM	594	14				x			
E/Mouse/IM	305	21				x			
E/Human/IM	417	14				x			
		21				x			
	686	14				x			
		21				x			
		90		x					
Count			1	2	0	27	3	1	0

S3 CD4

CD4									
Group (Adenoviral Species/Host Species/Administration Route)	Paper	Days post inoculation	Absolute Peaking Evidence (<10)	Strong Peaking Evidence (10-6)	Slight Peaking Evidence (2-6)	No Evidence	Slight Saturating Evidence (2-6)	Strong Saturating Evidence (10-6)	Strong Saturating Evidence (<10)
B/Mouse/IM	1201	14				x			
B/Human/IM	309	28	x						
		28		x					
C/Monkey/IM	1474	8				x			
		8				x			
D/Mouse/IM	924	14				x			
Count			1	1	0	4	0	0	0

S4 CD8

Group (Adenoviral Species/Host Species/Administration Route)	Paper	Days post inoculation	Absolute Peaking Evidence (<10)	Strong Peaking Evidence (10-6)	Slight Peaking Evidence (2-6)	No Evidence	Slight Saturating Evidence (2-6)	Strong Saturating Evidence (10-6)	Strong Saturating Evidence (<10)
B/Mouse/IM	1201	14				x			
	1269	56				x			
	1492	14	x						

B/Mouse/SQ	555	14				x				
		21				x				
		28				x				
		35				x				
		70				x				
		23				x				
		70				x				
B/Human/IM	309	28	x							
		28			x					
C/Mouse/IM	461	7				x				
		14			x					
		7	x							
		14				x				
	1039	8				x				
		8				x				
	1492	14	x							
	3018	7	x							
		15	x							
		30				x				
C/Mouse/SQ	555	14	x							
		21	x							
		28	x							
		35			x					
		70				x				
		23				x				
		70				x				
		14				x				
		21				x				
		28		x						
		35	x							
		70				x				
		23				x				
		70				x				
	2980	60				x				
C/Monkey/IM	1474	8				x				
		8				x				
D/Mouse/IM	924	14				x				
D/Mouse/SQ	555	14				x				

		21				x			
		28				x			
		35				x			
		70				x			
		23				x			
		70				x			
E/Mouse/IM	2919	10			x				
		20				x			
E/Mouse/SQ	555	14							
		21							
		28			x				
		35							
		70							
		23							
		70							
G/Mouse/SQ	555	14							
		21	x						
		28			x				
		35		x					
		70		x					
		23				x			
		70				x			
Unknown(sAd16)/ Mouse/SQ	555	14				x			
		21				x			
		28				x			
		35				x			
		70				x			
		23				x			
		70			x				
Count			11	3	7	42	0	0	0

S5 CD4 IFN+%

CD4 IFN									
Group (Adenoviral Species/Host Species/Administration	Paper	Days post inoculation	Absolute Peaking Evidence (<10)	Strong Peaking Evidence (10-6)	Slight Peaking Evidence (2-6)	No Evidence	Slight Saturating Evidence (2-6)	Strong Saturating Evidence (10-6)	Strong Saturating Evidence (<10)

Route)									
B/Human/IM	309	28		x					
	441	28	x						
Count			1	1	0	0	0	0	0

S6 CD8 IFN+%

CD8 IFN									
Group (Adenoviral Species/Host Species/Administration Route)	Paper	Days post inoculation	Absolute Peaking Evidence (<10)	Strong Peaking Evidence (10-6)	Slight Peaking Evidence (2-6)	No Evidence	Slight Saturating Evidence (2-6)	Strong Saturating Evidence (10-6)	Strong Saturating Evidence (<10)
B/Human/IM	309	28				x			
	441	28	x						
E/Mouse/IM	1801	10				x			
Count			1	0	0	2	0	0	0

S7 CD4 TNF+%

CD4 TNF									
Group (Adenoviral Species/Host Species/Administration Route)	Paper	Days post inoculation	Absolute Peaking Evidence (<10)	Strong Peaking Evidence (10-6)	Slight Peaking Evidence (2-6)	No Evidence	Slight Saturating Evidence (2-6)	Strong Saturating Evidence (10-6)	Strong Saturating Evidence (<10)
B/Human/IM	309	28			x				
	441	28				x			
Count			0	0	1	1	0	0	0

S8 CD8 TNF+%

CD8 TNF									
Group (Adenoviral Species/Host Species/Admi	Paper	Days post inoculation	Absolute Peaking Evidence (<10)	Strong Peaking Evidence (10-6)	Slight Peaking Evidence (2-6)	No Evidence	Slight Saturating Evidence (2-6)	Strong Saturating Evidence (10-6)	Strong Saturating Evidence (<10)

nistration Route)									
B/Human/IM	441	28			x				
Count			0	0	1	0	0	0	0

S9 CD4 IL2+%

CD4 IL2									
Group (Adenoviral Species/Host Species/Administration Route)	Paper	Days post inoculation	Absolute Peaking Evidence (<10)	Strong Peaking Evidence (10-6)	Slight Peaking Evidence (2-6)	No Evidence	Slight Saturating Evidence (2-6)	Strong Saturating Evidence (10-6)	Strong Saturating Evidence (<10)
B/Human/IM	309	28				x			
	441	28	x						
Count			1	0	0	1	0	0	0

S10 CD8 IL2+%

CD8 IL2									
Group (Adenoviral Species/Host Species/Administration Route)	Paper	Days post inoculation	Absolute Peaking Evidence (<10)	Strong Peaking Evidence (10-6)	Slight Peaking Evidence (2-6)	No Evidence	Slight Saturating Evidence (2-6)	Strong Saturating Evidence (10-6)	Strong Saturating Evidence (<10)
B/Human/IM	441	28			x				
Count			0	0	1	0	0	0	0

S11 CD4 IL17+%

CD4 IL17									
Group (Adenoviral Species/Host Species/Administration Route)	Paper	Days post inoculation	Absolute Peaking Evidence (<10)	Strong Peaking Evidence (10-6)	Slight Peaking Evidence (2-6)	No Evidence	Slight Saturating Evidence (2-6)	Strong Saturating Evidence (10-6)	Strong Saturating Evidence (<10)
B/Human/IM	309	28			x				

Count			0	0	1	0	0	0	0

S12 Virus Neutralisation Titre

Virus Neutralisation Titre									
Group (Adenoviral Species/Host Species/Administration Route)	Paper	Days post inoculation	Absolute Peaking Evidence (<10)	Strong Peaking Evidence (10-6)	Slight Peaking Evidence (2-6)	No Evidence	Slight Saturating Evidence (2-6)	Strong Saturating Evidence (10-6)	Strong Saturating Evidence (<10)
B/Mouse/SQ	2030	14				x			
	1269	56					x		
C/Mouse/IM	461	7				x			
		14				x			
	574	7				x			
		21				x			
		35	x						
		49				x			
		63				x			
		77				x			
		119			x				
		161				x			
	669	28				x			
	1039	8				x			
C/Mouse/SQ	2505	10				x			
		14				x			
C/Cattle/IM	2841	7						x	
D/Human/IM	594	14				x			
D/Rat/IM	594	28	x						
E/Mouse/SQ	2030	14				x			
E/Human/IM	686	14				x			
		21				x			
Count			2	0	1	17	1	1	0

Supplementary S1a, Vectors by Paper

Paper Number	Present Paper Name	Vector(s)
140 [15]	Use of ChAd3-EBO-Z Ebola virus vaccine in Malian and US adults, and boosting of Malian adults with MVA-BN-Filo: a phase 1, single-blind, randomised trial, a phase 1b, open-label and double-blind, dose-escalation trial, and a nested, randomised, double-blind, placebo-controlled trial	ChAd3
249 [16]	A Monovalent Chimpanzee Adenovirus Ebola Vaccine Boosted with MVA	ChAd3
305[17]	Characterization of T-Cell Responses to Conserved Regions of the HIV-1 Proteome in BALB/c Mice	ChAdV63
309 [18]	The novel tuberculosis vaccine, AERAS-402, is safe in healthy infants previously vaccinated with BCG, and induces dose-dependent CD4 and CD8T cell responses	Ad35
417 [19]	Clinical assessment of a novel recombinant simian adenovirus ChAdOx1 as a vectored vaccine expressing conserved Influenza A antigens	ChAdOx1
441 [20]	A phase 1b randomized, controlled, double-blinded dosage-escalation trial to evaluate the safety, reactogenicity and immunogenicity of an adenovirus type 35 based circumsporozoite malaria vaccine in Burkinabe healthy adults 18 to 45 years of age	Ad35
461 [21]	Beta-defensin 2 enhances immunogenicity and protection of an adenovirus-based H5N1 influenza vaccine at an early time	Ad5
467 [22]	Randomized, placebo-controlled trial to assess the safety and immunogenicity of an adenovirus type 35-based circumsporozoite malaria vaccine in healthy adults	Ad35
555[23]	Comparative analysis of the magnitude, quality, phenotype, and protective capacity of simian immunodeficiency virus gag-specific CD8+ T cells following human-, simian-, and chimpanzee-derived recombinant adenoviral vector immunization	Ad35, sAd11, sAd16, ChAd3, Ad5, Ad28, ChAd63
574 [24]	Recombinant adenovirus expressing type Asia1 foot-and-mouth disease virus capsid proteins induces protective immunity against homologous virus challenge in mice	Ad5
578 [25]	Ad35 and ad26 vaccine vectors induce potent and cross-reactive antibody and T-cell responses to multiple filovirus species	Ad35, Ad26
594[26]	First-in-human evaluation of the safety and immunogenicity of a recombinant adenovirus serotype 26 HIV-1 Env vaccine (IPCAVD 001)	Ad26
633 [27]	A phase I double blind, placebo-controlled, randomized study of a multigenic HIV-1 adenovirus subtype 35 vector vaccine in healthy uninfected adults	Ad35
669 [28]	Impact of preexisting adenovirus vector immunity on immunogenicity and protection conferred with an adenovirus-based H5N1 influenza vaccine	Ad5
686 [29]	Clinical assessment of a recombinant simian adenovirus ChAd63: a potent new vaccine vector	ChAd63
744 [30]	A novel alphavirus replicon-vectored vaccine delivered by adenovirus induces sterile immunity against classical swine fever	Ad5
924 [31]	TLR4 Ligands Augment Antigen-Specific CD8+ T Lymphocyte Responses Elicited by a Viral Vaccine Vector	Ad26
936 [32]	A Candidate H1N1 Pandemic Influenza Vaccine Elicits Protective Immunity in Mice	Ad5
1039[33]	Enhanced protection against Ebola virus mediated by an improved adenovirus-based vaccine	Ad5
1201 [34]	Impact of Recombinant Adenovirus Serotype 35 Priming versus Boosting of a Plasmodium falciparum Protein: Characterization of T- and B-Cell Responses to Liver-Stage Antigen 1	Ad35
1269 [35]	Increased immunogenicity of recombinant Ad35-based malaria vaccine through formulation with aluminium phosphate adjuvant	Ad35
1343 [36]	Recombinant low-seroprevalent adenoviral vectors Ad26 and Ad35 expressing the respiratory syncytial virus (RSV) fusion protein induce protective immunity against RSV infection in cotton rats	Ad26
1474 [37]	A novel adenovirus type 6 (Ad6)-based hepatitis C virus vector that overcomes preexisting anti-ad5 immunity and induces potent	Ad5, Ad6

	and broad cellular immune responses in rhesus macaques	
1492 [38]	Immunogenicity and Protection of a Recombinant Human Adenovirus Serotype 35-Based Malaria Vaccine against Plasmodium yoelii in Mice	Ad5, Ad35
1539 [39]	Induction of Protective Immunity to Anthrax Lethal Toxin with a Nonhuman Primate Adenovirus-Based Vaccine in the Presence of Preexisting Anti-Human Adenovirus Immunity	AdC7
1801[40]	Induction of CD8+ T cells to an HIV-1 antigen through a prime boost regimen with heterologous E1-deleted adenoviral vaccine carriers	AdC6
1877 [41]	Comparative immunogenicity in rhesus monkeys of DNA plasmid, recombinant vaccinia virus, and replication-defective adenovirus vectors expressing a human immunodeficiency virus type 1 gag gene	Ad5
2030 [42]	Novel, Chimpanzee Serotype 68-Based Adenoviral Vaccine Carrier for Induction of Antibodies to a Transgene Product	Ad5, ChAd63
2505 [43]	A replication-defective human adenovirus recombinant serves as a highly efficacious vaccine carrier	Ad5
2531 [44]	Isogenic adenoviruses type 5 expressing or not expressing the E1A gene: efficiency as virus vectors in the vaccination of permissive and non-permissive species	Ad5
2841 [45]	Efficacy of an adenovirus-vectored foot-and-mouth disease virus serotype A subunit vaccine in cattle using a direct contact transmission model	Ad5
2916[46]	Functionally inactivated dominant viral antigens of human cytomegalovirus delivered in replication incompetent adenovirus type 6 vectors as vaccine candidates	Ad6
2919 [47]	A prime-boost immunization regimen based on a Simian Adenovirus 36 vectored multi-stage malaria vaccine induces protective immunity in mice	sAd36
2980 [48]	Early life vaccination: Generation of adult-quality memory CD8+ T cells in infant mice using non-replicating adenoviral vectors	Ad5
3018 [49]	Inhibitory receptor expression on memory CD8 T cells following Ad vector immunization	Ad5

Supplementary S1b, Species and origin of vectors

Name	Species	Origin
Ad26	D	Human
Ad28	D	Human
Ad35	B	Human
Ad5	C	Human
Ad6	C	Human
AdC6	E	Simian
AdC7	E	Simian
Chad3	C	Simian
ChAd63	E	Simian
ChAdOx1	E	Simian
sAd11	G	Simian
sAd16	N.A.	Simian
sAd36	E	Simian
